# Optimization of an alum-anchored clinical HIV vaccine candidate

**DOI:** 10.1038/s41541-023-00711-0

**Published:** 2023-08-12

**Authors:** Kristen A. Rodrigues, Christopher A. Cottrell, Jon M. Steichen, Bettina Groschel, Wuhbet Abraham, Heikyung Suh, Yash Agarwal, Kaiyuan Ni, Jason Y. H. Chang, Parisa Yousefpour, Mariane B. Melo, William R. Schief, Darrell J. Irvine

**Affiliations:** 1 Koch Institute for Integrative Cancer Research, Massachusetts Institute of Technology, Cambridge, MA 02139 USA; 2 Harvard-MIT Health Sciences and Technology Program, Institute for Medical Engineering and Science, Massachusetts Institute of Technology, Cambridge, MA 02139 USA; 3 Ragon Institute of Massachusetts General Hospital, Massachusetts Institute of Technology and Harvard University, Cambridge, MA 02139 USA; 4 Consortium for HIV/AIDS Vaccine Development, The Scripps Research Institute, La Jolla, CA 92037 USA; 5 Department of Integrative Structural and Computational Biology, The Scripps Research Institute, La Jolla, CA 92037 USA; 6 Department of Immunology and Microbiology, The Scripps Research Institute, La Jolla, CA 92037 USA; 7 IAVI Neutralizing Antibody Center, The Scripps Research Institute, La Jolla, CA 92037 USA; 8 Department of Biological Engineering, Massachusetts Institute of Technology, Cambridge, MA 02139 USA; 9 Department of Chemical Engineering, Massachusetts Institute of Technology, Cambridge, MA 02139 USA; 10 Department of Materials Science and Engineering, Massachusetts Institute of Technology, Cambridge, MA 02139 USA; 11 Howard Hughes Medical Institute, Chevy Chase, MD 20815 USA

**Keywords:** Protein vaccines, Adjuvants

## Abstract

In the ongoing effort to develop a vaccine against HIV, vaccine approaches that promote strong germinal center (GC) responses may be critical to enable the selection and affinity maturation of rare B cell clones capable of evolving to produce broadly neutralizing antibodies. We previously demonstrated an approach for enhancing GC responses and overall humoral immunity elicited by alum-adjuvanted protein immunization via the use of phosphoserine (pSer) peptide-tagged immunogens that stably anchor to alum particles via ligand exchange with the alum particle surface. Here, using a clinically relevant stabilized HIV Env trimer termed MD39, we systematically evaluated the impact of several parameters relevant to pSer tag composition and trimer immunogen design to optimize this approach, including phosphate valency, amino acid sequence of the trimer C-terminus used for pSer tag conjugation, and structure of the pSer tag. We also tested the impact of co-administering a potent saponin/monophosphoryl lipid A (MPLA) nanoparticle co-adjuvant with alum-bound trimers. We identified MD39 trimer sequences bearing an optimized positively-charged C-terminal amino acid sequence, which, when conjugated to a pSer tag with four phosphates and a polypeptide spacer, bound very tightly to alum particles while retaining a native Env-like antigenicity profile. This optimized pSer-trimer design elicited robust antigen-specific GC B cell and serum IgG responses in mice. Through this optimization, we present a favorable MD39-pSer immunogen construct for clinical translation.

## Introduction

The human immunodeficiency virus/acquired immunodeficiency syndrome (HIV/AIDS) epidemic began more than 40 years ago. Despite the increasing availability of antiretroviral drugs, HIV remains a leading cause of death globally, with over 37 million people currently infected with HIV worldwide^1^. As a result, there is a significant unmet need for the development of an effective prophylactic HIV vaccine as a low-cost, facile solution to prevent infection^2^. Previous work has demonstrated that passive transfer of broadly neutralizing antibodies (bnAbs) capable of neutralizing diverse and mutated strains of HIV protects in non-human primate models of infection^3,4^. Further, a human trial of bnAb VRC01 infusion provided proof-of-concept that bnAb prophylaxis can be effective against recognized strains of the virus, suggesting that a bnAb-directed vaccine approach has the potential to prevent infection^5^. However, due to the diversity and high mutational capacity of the virus, the induction of bnAbs in the context of vaccination has remained a challenge^6–8^.

Prolonged antigen persistence following acute infection has been associated with improved immune responses^9–11^. Motivated by this observation, recent work has shown that the kinetics of antigen and adjuvant exposure in draining lymph nodes also significantly influences the immune response to vaccination: For example, immunization approaches providing sustained delivery of HIV antigens over 2 weeks (via repeated injections or implanted osmotic pumps) resulted in increased germinal center (GC) B cell, follicular helper T cell (Tfh), and serum antibody responses compared to traditional bolus approaches in mice^9^. In non-human primates, these sustained delivery approaches enhanced GC B cell responses, recruiting significantly more antigen-specific B cell clones to the GC reaction and dramatically increasing the induction of autologous tier 2 neutralizing antibody responses to a stabilized HIV Env trimer immunogen^10,12,13^. In an attempt to achieve similar effects on the immune response using more practical immunization regimens, we recently investigated a strategy to stably bind antigen to aluminum hydroxide (alum) particles, inspired by prior work showing that phosphorylated proteins bind tightly to aluminum hydroxide via ligand exchange reactions between phosphates and surface hydroxyls on alum particles^14,15^: short peptide tags composed of consecutive phosphoserines (pSer) were site-specifically attached to immunogens to serve as affinity tags for binding antigens in an oriented manner to alum particles^16^. While physically adsorbed antigens were rapidly released from alum following injection in vivo, pSer-tagging resulted in sustained release of antigen-alum complexes from the injection site, leading to enhanced antigen-specific GC B cell responses, serum antibody titers and increased development of long-lived bone marrow plasma cells for both HIV^16,17^ and SARS-CoV-2 antigens^18^.

**Fig. 1:**
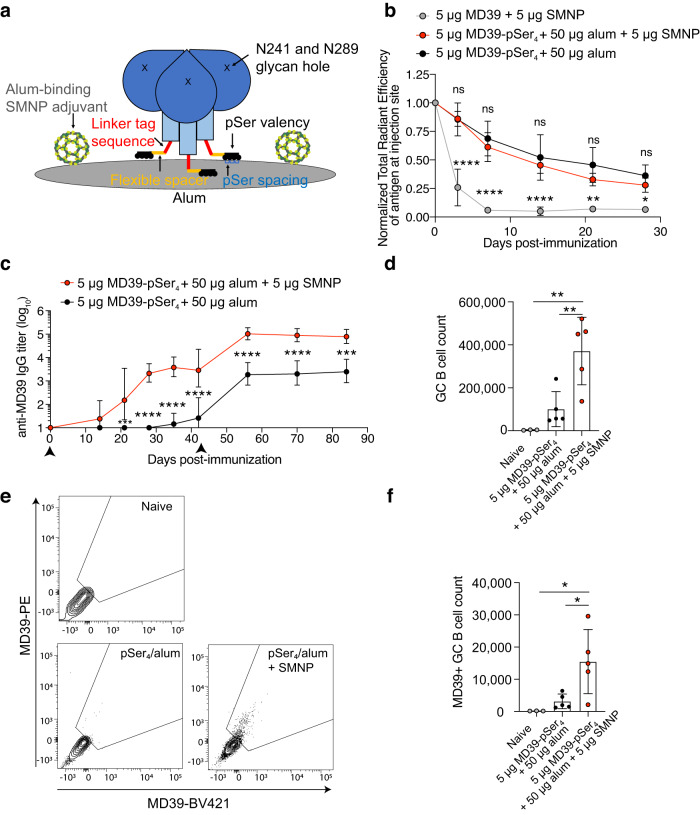
Combining MD39-pSer with alum-binding saponin nanoparticle co-adjuvant enhances humoral responses. **a** Schematic overview of parameters optimized for pSer-tagged trimers, including phosphoserine valency, immunogen linker tag sequence, pSer spacing, flexible linker spacer sequence, and effects of adding co-adjuvants to alum. **b** BALB/c mice (*n* = 5–11 animals/group) were immunized as indicated with fluorescently labeled antigen, and the fluorescence at the injection site was quantified longitudinally. Values plotted are means ± standard deviation. Statistical significance was determined by two-way ANOVA followed by Tukey’s multiple comparison test. **c** Mice (*n* = 5 animals/group) were immunized as indicated, and serum IgG responses were assessed longitudinally by ELISA. Arrows indicate immunizations. Values plotted are geometric means ± geometric standard deviation. Statistical significance was determined by two-way ANOVA followed by Tukey’s multiple comparison test. **d** Mice were immunized as indicated, and germinal center (GC) B cell responses in draining inguinal lymph nodes were analyzed by flow cytometry 14 days post-immunization. **e** Shown are representative flow cytometry gating plots of MD39-specific GC B cell analysis, plotted in (**f**). Values plotted are means ± standard deviation. Statistical significance was determined by one-way ANOVA followed by Tukey’s multiple comparison test. ns *p* > 0.05, *p < 0.05, **p < 0.01, ***p < 0.001, ****p < 0.0001.

Given these promising results, we sought to position the pSer-antigen/alum technology for potential clinical translation with a hyperstabilized HIV Env SOSIP trimer termed MD39^19,20^, which is a current clinical candidate as a potential “polishing” immunogen in sequential immunization regimens aiming to elicit bnAbs in humans^21^. Our prior proof-of-concept studies were performed using Env trimers modified with C-terminal His-tags capped by a cysteine for chemical attachment of pSer tags at the base of the trimer^16^. However, His-tags are immunogenic and undesirable for clinical vaccine products^22^. We also hypothesized that the design of the pSer tag itself might be optimized in terms of composition, phosphate valency, or phosphate spacing to mediate maximal alum binding. Thus, here we explored the design of the immunogen and pSer tag to maximize on-target, vaccine-relevant responses and minimize undesired responses to vaccine-irrelevant epitopes (Fig. 1a). We first investigated the impact of phosphate valency on the in vitro alum binding of MD39 as well as in vivo humoral immune responses, and tested the impact of combining alum delivery with a promising saponin nanoparticle-based adjuvant, SMNP^23^. We subsequently screened linkers expressed on the immunogen to replace the polyhistidine purification sequence used in our preclinical immunization studies, analyzing humoral responses to a set of downselected constructs that behaved favorably in vitro. The immunogen construct was further polished by filling a glycan hole^20^. Finally, we assessed the impact of phosphate spacing in the pSer tag and the composition of the linker. Together, these studies identified a final MD39-pSer optimized for potential further clinical development.

## Results

### Synergistic immune priming by combining MD39-pSer/alum with an alum-binding saponin/TLR-4 agonist nanoparticle adjuvant

In our initial proof-of-concept studies, we established a strategy to anchor stabilized HIV Env trimers to alum particles by chemically conjugating pSer peptide tags at the base of the trimer (Fig. 1a). pSer-tagged trimers are prepared by the reaction of a short maleimide-functionalized phosphoserine-containing peptide to a free cysteine introduced at the C-terminus of the MD39 gp140 protomers (Supplementary Table 1). In order to further boost responses to alum-anchored trimers, we investigated the effect of adding a nanoparticle adjuvant which is also capable of undergoing ligand exchange with alum. SMNP (Saponin/MPLA nanoparticle) is an ISCOMs-like nanoparticle ~40 nm in diameter comprised of saponin and the Toll-like receptor (TLR)-4 agonist monophosphoryl lipid A (MPLA) self-assembled with lipids and cholesterol^23^, which we previously showed could bind stably to alum in concert with pSer-tagged immunogens via ligand exchange between the phospholipid headgroups of the particle and alum^18^. We observed near-complete retention of SMNP on alum after 24-h incubation in phosphate buffer containing 10% mouse serum, regardless of the initial loading of MD39-pSer_4_ (MD39 trimer tagged with a peptide tag containing 4 phosphoserine residues, Supplementary Fig. 1a), consistent with our previous studies of pSer-tagged SARS-CoV-2 S receptor binding domain immunogens^18^. We next immunized mice with fluorescently labeled MD39-pSer_4_ bound to alum and tracked the clearance of antigen from the injection site using whole-animal fluorescence imaging. In these experiments, the addition of SMNP to the alum/MD39-pSer_4_ formulation did not substantially alter antigen drainage kinetics; both groups combining alum with the pSer-conjugated trimer showed much slower clearance of antigen from the injection site than the soluble MD39 trimer/SMNP comparator group (Fig. 1b). To assess the impact of SMNP as a co-adjuvant on humoral responses, we immunized mice with MD39-pSer_4_/alum combined with SMNP and boosted the animals at 6 weeks, tracking serum anti-MD39 IgG antibody responses longitudinally by ELISA. The addition of SMNP markedly improved antibody responses, leading to seroconversion of all animals post-prime (Fig. 1c). Further, SMNP significantly enhanced GC B cell responses (Fig. 1d–f, Supplementary Fig. 1b), inducing striking increases in MD39-specific GC B cell responses primed by MD39-pSer_4_/alum immunizations. Thus, SMNP enhances multiple facets of humoral immune responses following MD39-pSer_4_/alum vaccination.

### Role of pSer valency in modulating alum binding, antigen clearance rates in vivo, and vaccine immunogenicity

**Fig. 2:**
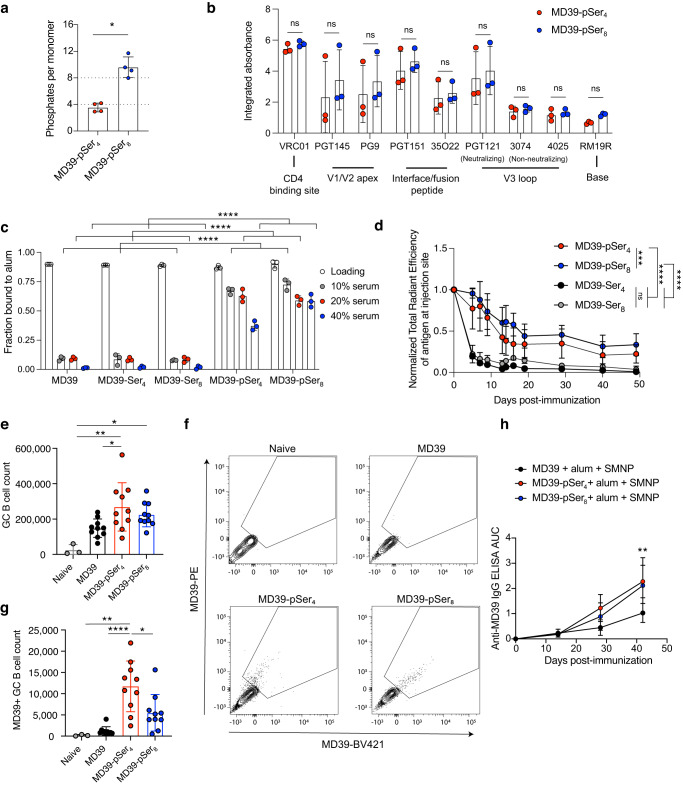
Phosphoserine valency modulates immunogen binding to alum and humoral responses to immunization. **a** MD39 conjugated to peptides containing 4 or 8 phosphoserines (MD39-pSer_4_, MD39-pSer_8_) was assayed for phosphates by a malachite green assay. **b** Antigenicity profiling of MD39-pSer_4_ and MD39-pSer_8_ on alum. Shown are area-under-the-curve values for trimer binding vs. antibody concentration. **c** pSer-conjugated, Ser-conjugated, or unmodified MD39 trimers were mixed with alum, and the fraction of protein bound to alum was assessed after a 24-h incubation in varying percentages of mouse serum at 37 °C. **d** BALB/c mice (*n* = 3 animals/group) were immunized with 10 µg fluorescently labeled MD39 plus alum, and injection site fluorescence was quantified longitudinally. **e** Mice (*n* = 3–10 animals/group) were immunized with 10 µg unmodified, pSer_4_−, or pSer_8_-conjugated MD39 and 100 µg alum, and antigen-specific germinal center (GC) B cell responses in draining inguinal lymph nodes were analyzed by flow cytometry 14 days post-immunization. **f**, **g** Shown are MD39-specific GC B cell counts and staining plots. **h** BALB/c mice (*n* = 5 animals/group) were immunized with 5 µg unmodified, pSer_4_- or pSer_8_-conjugated MD39 and 50 µg alum plus 5 µg SMNP, and serum IgG responses were assessed by ELISA. Statistical significance was determined by the Mann–Whitney *U* test for (**a**) and (**b**), one-way ANOVA for (**c**, **e**, and **g**) followed by Tukey’s multiple comparison test, and two-way ANOVA for (**d**) and (**h**) followed by Tukey’s multiple comparison test. Values plotted are means ± standard deviation. ns *p* > 0.05, *p < 0.05, **p < 0.01, ***p < 0.001, ****p < 0.0001.

Since the interactions between pSer-antigens and alum are mediated by the phosphate groups on pSer linkers, we next investigated the impact of pSer valency (number of phosphates in the affinity tag) on the stability of trimer binding to alum (Supplementary Fig. 2a). We previously found that tags containing four phosphoserines (pSer_4_) were effective at stably binding monomeric and trimeric immunogens to alum^16,18^. However, we hypothesized there could be a benefit to further increasing the valency of the pSer tag. To investigate this, peptide tags containing 4 or 8 pSer residues were synthesized, and the impact of pSer valency on alum binding in vitro was investigated. Measurement of the mean number of phosphates per protomer using a malachite green assay revealed 3.5 and 9.6 phosphates per MD39 protomer for MD39-pSer_4_ and MD39-pSer_8_, respectively, near the expected ~4 and ~8 phosphates per promoter (Fig. 2a). To confirm that phosphoserine-tagged trimers retained structural integrity upon binding to alum, we used a modified sandwich ELISA approach to probe the antigenicity profiles of the constructs: pSer-conjugated MD39 was captured on alum-coated plates, and the immobilized immunogen was probed for binding to serial dilutions of a panel of neutralizing and non-neutralizing monoclonal antibodies. This analysis revealed comparable antigenicity profiles for MD39-pSer_4_ and -pSer_8_ on alum for the CD4 binding site, V1/V2 apex, interface/fusion peptide, and neutralizing V3 loop epitopes (Fig. 2b, Supplementary Fig. 2b). Importantly, both pSer-conjugated trimers exhibited low binding to antibodies targeting non-neutralizing V3 loop epitopes and the trimer base. The latter is expected if anchoring the trimers to alum sterically obscures the base of the immunogen, which is important since the base of soluble HIV Env trimers is known to be a highly immunogenic surface that can be immunodominantly targeted by the antibody response and irrelevant for protection^24^. Next, we assessed the stability of alum binding for the pSer-tagged immunogens. MD39 trimers were incubated with alum for 30 min in tris-buffered saline to allow adsorption to the alum particles, followed by incubation for 24 h in increasing concentrations of mouse serum at 37 °C to test binding stability. Unmodified MD39 showed poor retention on alum, with the majority of antigen desorbed following incubation with only 10% mouse serum (Fig. 2c). In comparison, the pSer-modified antigens exhibited much greater retention on alum in the presence of serum, and the higher phosphate valency MD39-pSer_8_ showed greater retention on alum than MD39-pSer_4_.

Next, we investigated whether these differences in alum binding observed in vitro translated to differential antigen clearance kinetics in vivo. Mice were immunized subcutaneously (s.c.) at the tail base with fluorophore-labeled MD39-pSer_4_ or MD39-pSer_8_ mixed with alum or control serine-tagged MD39-Ser_4_ or MD39-Ser_8_ and alum, and the kinetics of antigen clearance from the injection site were tracked longitudinally by whole animal fluorescence imaging. For the control Ser_4_- and Ser_8_-conjugated trimers that are incapable of ligand exchange-mediated binding to alum, we observed rapid antigen clearance from the immunization site, with only ~20% of the antigen remaining by 5 days post injection (Fig. 2d). By contrast, MD39-pSer_4_ and MD39-pSer_8_ both exhibited significantly increased persistence at the injection site, with a steady decay over ~3 weeks followed by slower clearance at later times; MD39-pSer_8_/alum cleared slightly slower than MD39-pSer_4_/alum (*p* < 0.001, Fig. 2d).

To assess whether these differences in antigen drainage kinetics impact the resulting humoral immune response, we immunized mice with MD39, MD39-pSer_4,_ or MD39-pSer_8_ mixed with alum and carried out flow cytometry analysis of GC B cell responses in draining inguinal lymph nodes at 14 days post-immunization. Although both unmodified and pSer-tagged MD39 elicited substantial total GC B cell responses (Fig. 2e), trimer-binding GC B cells were barely detectable at this time point for immunization with non-tagged trimer and alum (Fig. 2f, g). Notably, the absolute magnitude of trimer-specific GC B cells was significantly greater for MD39-pSer_4_/alum immunization and trended toward more than double the response elicited by MD39-pSer_8_/alum (Fig. 2f, g). Next, we immunized mice with MD39, MD39-pSer_4,_ or MD39-pSer_8_ mixed with alum plus SMNP to maximize the humoral response, tracking the serum anti-MD39 IgG antibody response (Fig. 2h). The serum IgG titers elicited by MD39-pSer_8_/alum immunization were comparable to MD39-pSer_4_/alum. Thus, we focused on a 4-valent phosphoserine tag for subsequent studies.

### Optimizing the C-terminal sequence of MD39 trimers for pSer tag conjugation

**Fig. 3:**
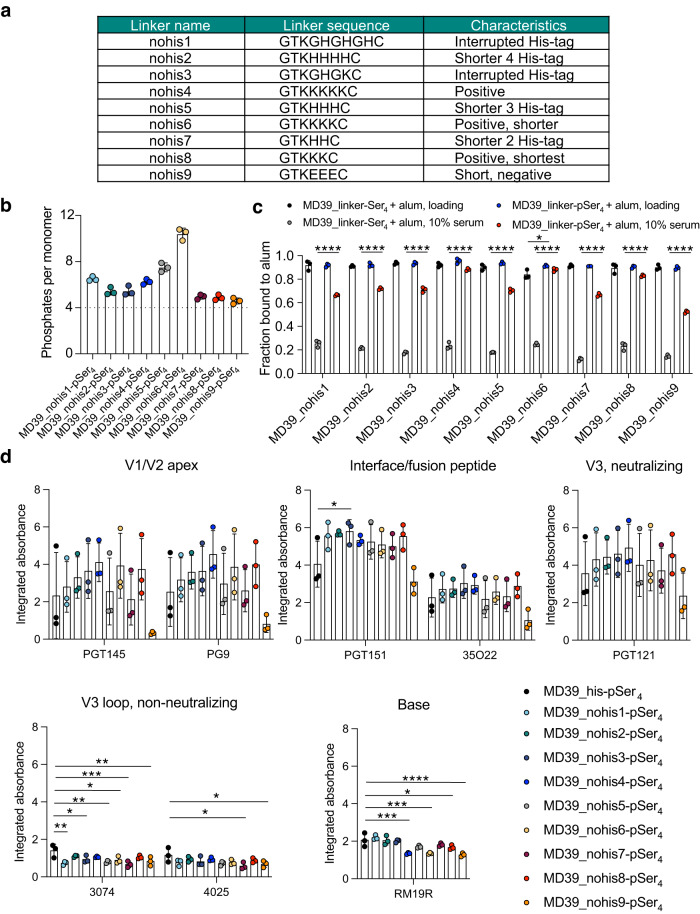
MD39 constructs containing alternate linkers retain alum binding properties and antigenicity profile when conjugated to pSer. **a** Table of alternate linker sequences and their attributes. **b** MD39 constructs containing these linkers were expressed, conjugated to pSer_4_ peptides, and assayed for phosphates per protein by a malachite green assay. Values plotted are means ± standard deviation. **c** pSer-conjugated or unmodified MD39 constructs were mixed with alum, and the fraction of protein bound to alum was assessed before (“Loading”) and after incubation for 24 h in 10% mouse serum at 37 °C. Values plotted are means ± standard deviation. Statistical significance was determined by one-way ANOVA followed by Tukey’s multiple comparison test. **d** Antigenicity profiling of MD39-pSer_4_ on alum compared to MD39_his-pSer_4_. Shown are the area under individual binding curves normalized to the fraction of protein loaded on alum. Values plotted are means ± standard deviation. Statistical significance was determined by one-way ANOVA followed by Dunnett’s multiple comparison test. ns *p* > 0.05, *p < 0.05, **p < 0.01, ***p < 0.001, ****p < 0.0001.

Our preclinical studies to this point employed trimer proteins bearing a C-terminal His-tag sequence just before the cysteine residue used for pSer peptide coupling. While the incorporation of an N- or C-terminal polyhistidine tag is commonly used to facilitate the purification of recombinant proteins using immobilized metal ion affinity chromatography for preclinical studies, His-tags can be immunogenic^22^. This response has the potential to be immunodominant, such that B cells that recognize the polyhistidine linker could outcompete those which recognize more relevant antigen-specific epitopes. To replace the polyhistidine linker, we synthesized MD39 constructs incorporating a panel of 9 alternate cysteine-terminated C-terminal spacers varying in length and overall charge and screened these proteins for pSer conjugation, alum binding, and antigenicity profiles (Fig. 3a). Each of these immunogens was conjugated to pSer_4_ tags, and the degree of labeling was assessed by a malachite green assay. Several of the constructs showed over-labeling with significantly more than the target ~4 mean phosphates per protomer, including MD39_nohis5 and MD39_nohis6, suggesting that the maleimide-pSer tag was coupling to additional residues (e.g., lysine primary amines) and not just the terminal free cysteine (Fig. 3b)^25,26^. To assess the impact of the immunogen linker on alum binding behavior, pSer_4_-conjugated or unmodified MD39 constructs were mixed with alum, and the fraction of protein bound to alum was assessed before and after incubation for 24 h in 10% mouse serum at 37 °C. Several of the trimers bearing “nohis” linkers exhibited very stable binding to alum, with up to ~90% of the pSer-modified antigen remaining bound after serum incubation (e.g., MD39_nohis4, MD39_nohis6, MD39_nohis8, Fig. 3c). By contrast MD39_nohis9-pSer_4_ demonstrated weaker retention on alum compared to other constructs (Fig. 3c). Excluding the over-labeled MD39_nohis6-pSer_4_, MD39_nohis4-pSer_4_ and MD39_nohis8-pSer_4_ exhibited the best retention on alum (Fig. 3c) and showed more stable binding to alum in the presence of serum than our original MD39_his-pSer_4_ construct (Supplementary Fig. 3a). To assess whether the alum-bound MD39_nohis pSer-conjugates were structurally intact when bound to alum, we evaluated the antigenicity profiles of the alum-bound constructs. While most constructs retained major epitopes indicative of structurally intact antigen, construct MD39_nohis9-pSer_4_ in particular exhibited substantially lower binding of antibodies targeting the V1/V2 apex, interface/fusion peptide, and neutralizing V3 epitopes, suggesting partial unfolding of this trimer construct upon pSer-mediated alum binding (Fig. 3d, Supplementary Fig. 3b). Interestingly, the non-histag-containing MD39 constructs 4, 6, 8, and 9 showed lower binding of the trimer base-specific mAb RM19R, suggesting that they achieved more effective blocking of the trimer base when immobilized on alum. Considering the alum binding data, constructs MD39_nohis4 and MD39_nohis8 appeared most promising given strong V1/V2 apex, interface/fusion peptide, and neutralizing V3 epitope binding while shielding non-neutralizing epitopes. As a result, we sought to move forward with in vivo investigation of humoral responses to these two constructs.

**Fig. 4:**
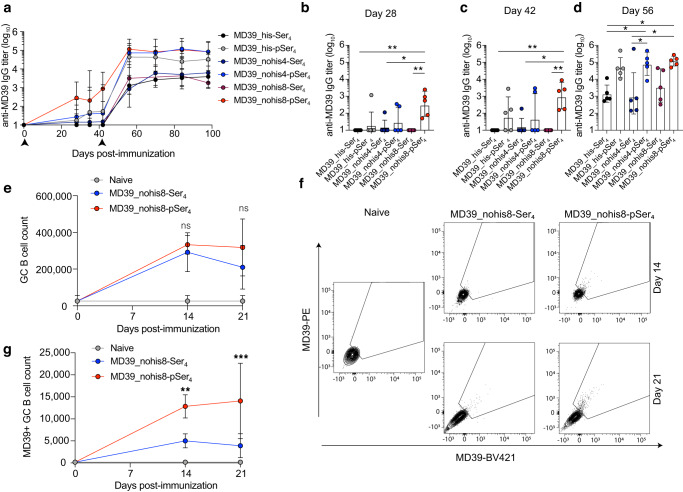
MD39 constructs containing alternate linkers conjugated to pSer elicit strong humoral immune responses. **a** BALB/c mice (*n* = 5 animals/group) were immunized with 10 µg Ser_4_- or pSer_4_-conjugated MD39 constructs and 100 µg alum and boosted with 5 µg Ser_4_- or pSer_4_-conjugated MD39 constructs and 50 µg alum with 5 µg SMNP at 6 weeks. Serum IgG responses were assessed longitudinally by ELISA. Arrows indicate immunizations. **b** Individual responses plotted for day 28. **c** Individual responses plotted for day 42. **d** Individual responses plotted for day 56. Values plotted are geometric means ± geometric standard deviation. Statistical significance was determined by two-way ANOVA followed by Tukey’s multiple comparison test. **e** Mice (*n* = 5 animals/group) were immunized with 5 µg pSer_4_- or Ser_4_-conjugated MD39_nohis8 construct and 50 µg alum with 5 µg SMNP, and germinal center (GC) B cell responses were assessed in the draining inguinal lymph nodes at day 14 and 21 post-immunization. **f** Shown are representative flow cytometry gating plots of MD39-specific GC B cell analysis, plotted in (**g**). Statistical significance was determined by two-way ANOVA followed by Tukey’s multiple comparison test. Values plotted are means ± standard deviation. ns *p* > 0.05, *p < 0.05, **p < 0.01, ***p < 0.001, ****p < 0.0001.

We first assessed the immunogenicity of these modified trimers in BALB/c mice compared to MD39 containing a polyhistidine linker. Mice were immunized with Ser_4_- or pSer_4_-conjugated trimers and alum and then boosted 6 weeks later with the same constructs combined with alum and SMNP (Fig. 4a). Post-prime, MD39_nohis8-pSer_4_ elicited significantly stronger responses compared to all other groups, with seroconversion observed in all animals immunized with MD39_nohis8-pSer_4_ vs. only 3 of 5 animals receiving histagged MD39 and 2 of 5 animals receiving MD39_nohis4-pSer_4_ (Fig. 4b, c). Two weeks post-boost, there was no significant difference in anti-MD39 serum IgG titers between the three pSer_4_-conjugated MD39 constructs, though MD39_nohis8-pSer_4_ elicited the most consistent high titers (Fig. 4d). Further, we assessed the proportion of the antibody response directed against the trimer base via ELISA assays using plate-bound trimer in the presence or absence of a base-blocking monoclonal antibody (Supplementary Fig. 4a), and found that MD39_nohis8-pSer_4_ elicited the lowest base response (Supplementary Fig. 4b). We previously showed that the phosphoserine anchoring peptides elicit no measurable antibody response^16^. Here, we separately tested whether the C-terminal sequences appended to the trimer prior to the pSer sequence elicited any antibody response and found no serum IgM or IgG responses against these linker peptides that were statistically significant over the background of naïve animals (Supplementary Fig. 5). We next assessed MD39-specific GC responses at days 14 and 21 post-immunization in mice immunized with Ser_4_- or pSer_4_-conjugated MD39_nohis8 combined with alum and SMNP. Flow cytometry analysis of draining inguinal lymph nodes revealed strong total GC responses in mice immunized with either control Ser_4_-trimers or alum-binding pSer_4_-conjugated trimers, but the pSer-tagged trimer elicited a significantly stronger antigen-specific GC B cell response that was sustained from day 14 to day 21 (Fig. 4e–g).

### Optimizing pSer tag design

**Fig. 5:**
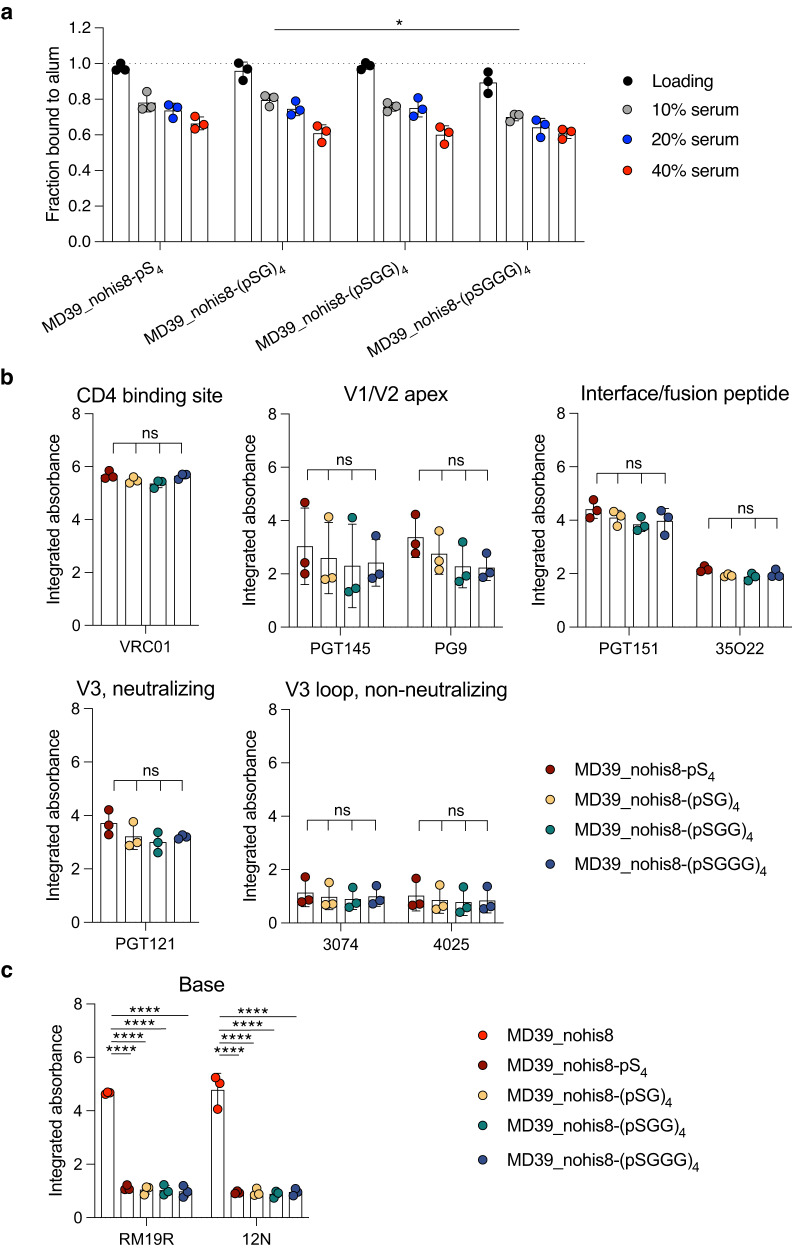
Glycine spacers between pSer residues do not significantly alter the physical properties of MD39-pSer. **a** MD39 was conjugated to pSer linkers containing 0–3 glycine residue spacers between pSer residues, and these constructs were mixed with alum. The fraction of protein bound to alum was assessed before (“Loading”) and after incubation for 24 h in varying amounts of mouse serum at 37 °C. Values plotted are means ± standard deviation. Statistical significance was determined by one-way ANOVA followed by Tukey’s multiple comparison test. **b**, **c** Antigenicity profiling of MD39-pSer_4_ constructs. Shown are the area under individual binding curves. Values plotted are means ± standard deviation. Statistical significance was determined by one-way ANOVA followed by Tukey’s multiple comparison test. ns *p* > 0.05, *p < 0.05, **p < 0.01, ***p < 0.001, ****p < 0.0001.

Although peptide tags comprised of four repeats of phosphoserine provided effective binding to alum, we hypothesized that separating phosphoserine groups with uncharged spacer residues might enable more efficient complexation of the pSer tag with the surface of alum particles and thereby further enhance the stability of trimer binding to alum. To test this idea, we screened additional pSer tags containing glycine amino acid spacers between pSer residues prepared by solid phase peptide synthesis using glycine repeats of 0 to 3 residues (pS_4_, (pSG)_4_, (pSGG)_4_, and (pSGGG)_4;_ Supplementary Fig. 6a). These tags were conjugated to MD39_nohis8, and the impact on alum binding and trimer antigenicity on alum was assessed. As shown in Fig. 5a, initial binding to alum in buffer was >90% for all 4 pSer tags, and they performed comparably well at promoting retention of the trimer on alum following serum exposure. Structural analysis of trimers bound to alum using neutralizing and non-neutralizing mAbs revealed a similar antigenicity profile for all trimers linked with all 4 pSer tag variants, and all 4 tags showed a similar low binding by trimer base-specific antibodies (Fig. 5b, c, Supplementary Fig. 6b). Based on these findings showing no further improvement by changing the peptide sequence, we moved forward with the pSer_4_ tag containing four sequential phosphoserines.

**Fig. 6:**
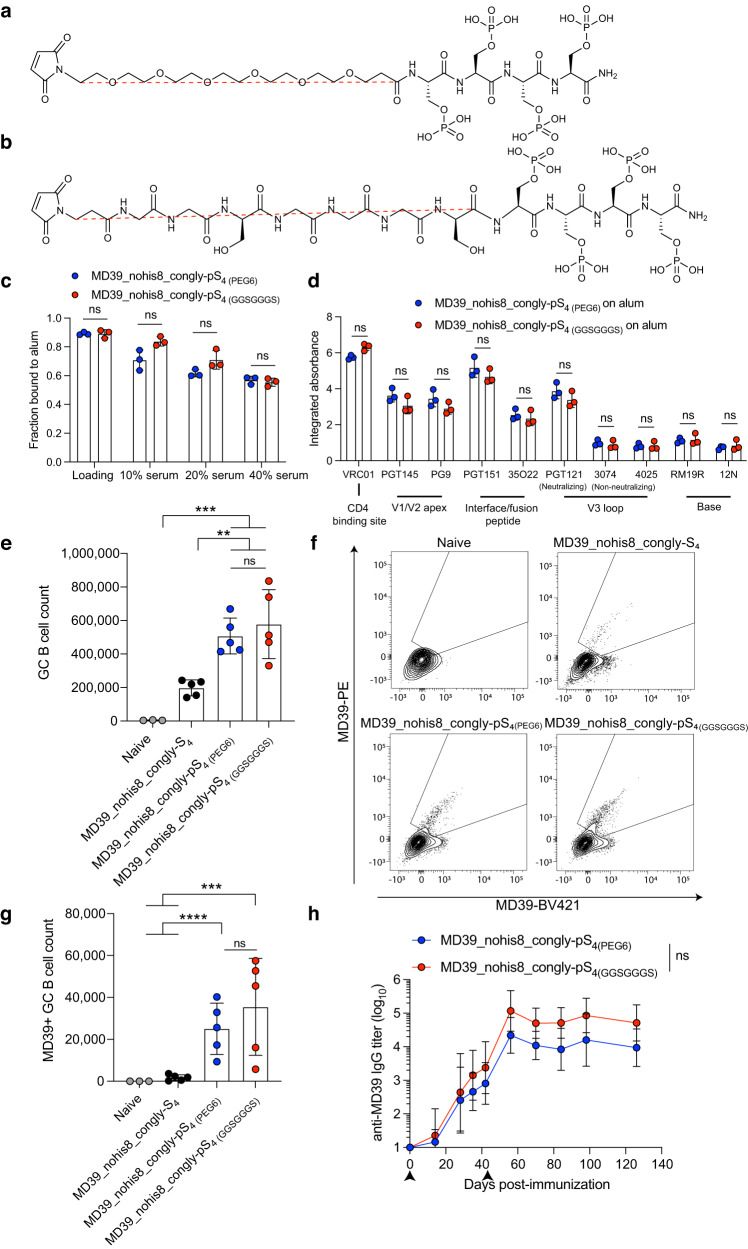
Replacing the 6-unit PEG spacer with a flexible glycine/serine spacer does not significantly alter the physical properties of MD39_nohis8_congly-pSer. Chemical structures of pSer linkers containing **a** 6-unit poly(ethylene glycol) (PEG_6_) or **b** glycine–glycine–serine–glycine–glycine–glycine–serine repeat (GGSGGGS) spacers. **c** pSer-conjugated MD39 constructs were mixed with alum, and the fraction of protein bound to alum was assessed before and after 24-h incubation in varying percentages of mouse serum at 37 °C. **d** Antigenicity profiling of MD39_nohis8_congly-pSer_4_ captured on alum. Shown are the area-under-individual binding curves. **e** BALB/c mice were immunized with 5 µg pSer_4_-conjugated MD39 constructs and 50 µg alum with 5 µg SMNP, and germinal center (GC) B cell responses in draining inguinal lymph nodes were analyzed by flow cytometry 14 days post-immunization. **f** Shown are representative flow cytometry gating plots of MD39-specific GC B cell analysis, plotted by MD39-specific GC B cell count in (**g**)**. h** Mice (*n* = 5 animals/group) were immunized with 10 µg pSer_4_-conjugated MD39 and 100 µg alum and boosted with 5 µg Ser_4_- or pSer_4_-conjugated MD39 and 50 µg alum with 5 µg SMNP at 6 weeks, and serum IgG responses were assessed longitudinally by ELISA. Arrows indicate immunizations. Values plotted are means ± standard deviation in (**c**–**f**) and geometric means ± geometric standard deviation in (**h**). Statistical significance was determined by the Mann–Whitney *U* test in (**c**) and (**d**), one-way ANOVA followed by Tukey’s multiple comparison tests in (**e**) and (**g**), and two-way ANOVA followed by Sidak’s multiple comparison tests in (**h**). ns *p* > 0.05, *p < 0.05, **p < 0.01, ***p < 0.001, ****p < 0.0001.

To further optimize the pSer tag design, we sought to replace the 6-unit polyethylene glycol (PEG_6_) spacer present in our original pSer tag design with a short glycine/serine spacer (GGSGGGS) of comparable fully-extended length (Fig. 6a, b), in order to reduce any potential for PEG-driven allergic reactions^27,28^ or off-target anti-PEG antibody responses, which have been previously reported for PEGylated drugs^29^. Glycine/serine repeat linkers are commonly used in engineered multidomain proteins and single chain variable fragments^30–32^ and are present in FDA-approved drugs that exhibit minimal immunogenicity^33,34^. Prior to testing this fully peptide-based pSer_4_ tag, we sought to fill an immunogenic glycan hole in the trimer immunogen at residues N241 and N289 by sequence modification to improve N-linked glycan processing. This glycan hole is a target for HIV strain-specific neutralizing responses that cannot mature to a broadly neutralizing antibody response^35,36^. This construct, MD39_nohis8_congly, showed the expected binding and antigenicity profile on alum when modified with phosphoserines (Supplementary Fig. 7a–c). We assessed the structural integrity of the glycan hole-filled trimer on alum by antigenicity ELISA, confirming that the antigen retains major neutralizing epitopes while shielding the trimer base comparably to the glycan hole-containing construct (Supplementary Fig. 7b, c). Further, GC and serum antibody responses were similar when comparing pSer_4_-conjugated trimers with or without the glycan hole filled, albeit with slightly lower total serum binding antibodies for the former, as expected since the congly trimer has less exposed protein surface (Supplementary Fig. 8). Based on these findings, the MD39_nohis8_congly construct was conjugated to the PEG_6_ pS_4 (PEG6)_ or GGSGGGS pS_4 (GGSGGGS)_ tags for biochemical and immune response analysis. Initial alum binding of trimer modified with the two different pSer tags was essentially identical (Fig. 6c, “loading”), and trimers tagged with the full peptide spacer trended towards slightly greater retention on alum when challenged with 10 or 20% serum (Fig. 6c). Antigenicity profiling revealed no significant difference between trimers captured on alum using either tag design, with comparable shielding of the trimer base for each spacer (Fig. 6d, Supplementary Fig. 9a). We then compared GC responses and serum antibody titers elicited by these two different pSer tag designs for MD39_nohis8_congly-pS_4_ combined with alum and SMNP. MD39_nohis8_congly modified with the two pSer tag types elicited comparable total GC B cell counts as well as comparable numbers of MD39-specific GC B cells, and these responses were significantly stronger than responses elicited by the control MD39_nohis8_congly-S_4_ trimer (Fig. 6e–g). We then compared the immunogenicity of the constructs, immunizing mice with Ser_4_- or pSer_4_-conjugated MD39 constructs and alum, followed by boosting with Ser_4_- or pSer_4_-conjugated MD39 constructs combined with alum and SMNP at 6 weeks. As shown in Fig. 6h, there was a trend toward increased serum IgG responses using the peptide-based linker vs. the PEG linker, though this did not reach statistical significance. While no anti-PEG or anti-GGSGGGS IgG responses were detected in either group at week 8 post-prime (week 2 post-boost), weak anti-PEG IgM responses were detected in the PEG linker group (Supplementary Fig. 10), and we detected no statistically significant IgM or IgG responses against the GGSGGS sequence above the background of naïve control mice (Supplementary Fig. 10).

Finally, we compared GC and antibody responses elicited by this optimized alum-anchored trimer vs. a protein nanoparticle form of the MD39 trimer, based on the fusion of MD39 with ferritin, to create a nanoparticle displaying 8 copies of MD39^37^. As shown in Supplementary Fig. 11a, b, ferritin-MD39 and MD39-pSer_4_/alum elicited similar total GC responses, but the alum-anchored trimer showed a trend toward increased antigen-binding GC B cell priming. Altogether, these data suggest that MD39_nohis8_congly-pS_4 (GGSGGGS)_ is a favorable construct for potential clinical translation.

## Discussion

Recent studies have demonstrated an important role in the timing of antigen and adjuvant delivery to lymph nodes during immunization, with prolonged vaccine exposure over ~2–4 weeks priming enhanced humoral immunity compared to bolus immunization in both mice and non-human primates^9,10,12^. This has motivated recent efforts to prolong vaccine exposure through technologies such as slow-release biodegradable hydrogels and microneedle skin patch vaccination^38–42^. These approaches have demonstrated stronger T cell and GC B cell responses, leading to improved affinity maturation, expanding the breadth, neutralization, and duration of antibody responses. While these approaches all appear promising, successful clinical translation of new delivery technologies is challenging due to the high bar for safety in vaccines and scalable manufacturing challenges. We recently developed an approach to achieve “extended dosing” effects using the traditional adjuvant alum by modifying antigens with a short tag containing pSer residues which anchor the antigen to alum through ligand exchange. This interaction promotes sustained drainage of antigen-alum complexes from the immunization site over the course of weeks, resulting in stronger antigen-specific GC B cell responses, neutralizing antibody titers, and increased development of long-lived bone marrow plasma cells for both HIV^16^ and SARS-CoV-2 antigens^18^. Ligand exchange between alum and phosphate-containing co-adjuvants enabled sustained co-delivery of antigens and co-adjuvants to synergistically enhance vaccine immunogenicity in mice and rhesus macaques, inducing neutralizing responses against SARS-CoV-2 variants.

Here we carried out a systematic study evaluating design parameters intrinsic to the pSer-antigen/alum approach, with the goal of optimizing a stabilized trimer design to maximally elicit on-target, vaccine-relevant responses. We tested the importance of pSer valency, immunogen linkers, and the composition of the pSer peptide tag and employed an alum-binding co-adjuvant SMNP to synergistically boost humoral immune responses. Consistent with our findings using a SARS-CoV-2 receptor binding domain antigen, the phosphate valency of pSer_4_ is sufficient to enhance humoral immune responses. We similarly observed a reduction in responses for pSer_8_, which may be due to altered antigen drainage kinetics. Interestingly, replacing the polyhistidine linker at the C-terminus of the trimer with a positively charged amino acid sequence significantly improved alum binding strength and trimer base shielding with improved immunogenicity in mice when conjugated to pSer_4_. Inter-pSer glycine spacer residues did not have a significant effect, suggesting the flexibility of the peptide promotes sufficient access of pSer phosphates to hydroxyls on the surface of alum for ligand exchange. Unexpectedly, we observed a slight improvement in immune responses when the PEG_6_ linker in the pSer tag was replaced with a GGSGGGS amino acid sequence. This may be due to alterations in the interaction of the linker sequence with the surface of alum particles or subtle changes in the linker organization at the trimer base. This optimized MD39-pSer/alum approach elicited robust antigen-specific GC B cell and serum IgG responses, maximizing humoral responses and minimizing off-target, vaccine-irrelevant responses to non-neutralizing epitopes.

Previous foundational work investigated the effect of phosphorylation on antigen adsorption and retention on alum, as well as its impact on immune responses^14,15,43,44^. These studies used protein-intrinsic phosphates or “zero-length” crosslinkers with phosphates. Here, we systematically modulated and evaluated parameters relevant to both the alum-binding tag and antigen, including phosphate valency and spacing, spacer sequence, and antigen linker design. Such modifications improved vaccine-elicited immune responses and helped to advance this platform toward potential clinical relevance.

In conclusion, through facile modification of immunogens with a short peptide linker, we observed striking enhancements in antibody and GC responses using alum, a well-established adjuvant, to achieve extended dosing in vaccination to prolong antigen availability to achieve stronger and more durable GC responses. This platform transforms alum, a low-cost FDA-approved vaccine adjuvant with a long clinical record of safety and modest efficacy when mixed with immunogens, into a more efficacious clinically relevant platform applicable to the development of vaccines targeting other established and emerging infectious diseases and cancer. Through co-anchoring of additional adjuvants, this approach may support the investigation of synergistic combinations of adjuvants to further advance vaccine development.

## Methods

### Phosphoserine peptide synthesis

pSer peptide linkers were synthesized using solid phase synthesis on low-loading TentaGel Rink Amide resin (0.2 meq/g, Peptides International)^16,18^. Briefly, the resin was deprotected with 20% piperidine (Sigma Aldrich) in dimethylformamide (DMF, Sigma Aldrich), and peptide couplings were performed with 4 equivalents of Fmoc-Ser(PO(OBzl)OH)-OH (Millipore Sigma) and 3.95 equivalents of hexafluorophosphate azabenzotriazole tetramethyl uronium (HATU) for 2 h at 25 °C in DCM and DMF (1:2). pSer residues were deprotected with 5% 1,8-diazabicyclo[5.4.0]undec-7-ene (DBU) in DMF. Double couplings were performed after the third residue. A Fmoc-protected 6-unit oligoethylene glycol linker (Peptides International) was then coupled to the peptide and subsequently deprotected and reacted with N-maleoyl-β-alanine (Sigma Aldrich). Completion of each deprotection and coupling step was confirmed by a ninhydrin test (Sigma Aldrich). pSer side chains were deprotected, and the peptide was cleaved from the resin in 95% trifluoroacetic acid (Sigma Aldrich), 2.5% H_2_O, and 2.5% triisopropylsilane (Sigma Aldrich) for 2.5 h at 25 °C. The product was precipitated in 4 °C diethyl ether (Sigma Aldrich) and dried under N_2_, then purified by HPLC on a C18 column (Agilent Zorbax 300SB-C18) using 0.1 M triethylammonium acetate buffer (Glen Research) in an acetonitrile gradient. The peptide mass was confirmed by matrix-assisted laser desorption/ionization-time of flight mass spectrometry.

For imaging experiments, a pSer_4_-AlexaFluor488 conjugate was synthesized as described for the pSer component of the linker, followed by deprotection and coupling to Fmoc-5-azido-pentanoic acid (Anaspec). The peptide was deprotected with 20% piperidine in dimethylformamide prior to cleavage from the resin in 95% trifluoroacetic acid, 2.5% H_2_O, and 2.5% triisopropylsilane for 2.5 h at 25 °C. The product was then precipitated in 4 °C diethyl ether, dried under N_2_, and purified by HPLC on a C18 column using 0.1 M triethylammonium acetate buffer in an acetonitrile gradient. The peptide mass was confirmed by matrix-assisted laser desorption/ionization-time of flight mass spectrometry. This pSer_4_-azide linker was reacted with one equivalent of AlexaFluor488-DBCO (Click Chemistry Tools) overnight at 4 °C in a Cu-free click reaction in PBS (pH 7.2–7.4) and subsequently purified by HPLC on a C18 column using 0.1 M triethylammonium acetate buffer in an acetonitrile gradient. The pSer peptide tags containing glycine spacers between pSer residues and glycine-glycine-serine-glycine-glycine-glycine-serine in place of the poly(ethylene glycol) spacer were prepared by solid phase peptide synthesis by Almac and characterized by HPLC and mass spectrometry.

### Antigen production and pSer conjugation

MD39 immunogens were synthesized by cloning genes encoding MD39 immunogens into pHLsec by Genscript and co-transfected with human furin in a pcDNA3.1 plasmid using a 2:1 trimer:furin DNA ratio with polyethylenimine into FreeStyle 293-F cells (ThermoFisher) and incubated for 6 days^20,45^. The cultures were centrifuged, and the supernatants containing MD39 were harvested and purified using a HisTrap HP column (Cytiva Life Sciences) with an AKTA FPLC system (Cytiva Life Sciences) for immunogens expressed with a polyhistidine linker and a 2G12 immunoaffinity column for MD39 immunogens without a polyhistidine linker. The immunogens were further purified by size-exclusion chromatography with an S200 Increase column (Cytiva Life Sciences) in TBS at a flow rate of 0.5 ml/min. Size exclusion chromatography multi-angle light-scattering (SECMALS, DAWN HELEOS II and Optilab T-rEX Wyatt Technology) was then used to confirm the immunogen molecular weight.

Immunogens expressed with a free terminal cysteine were reduced at 1 mg/ml with 10 molar equivalents of tris(2-carboxyethyl)phosphine (TCEP, ThermoFisher) and incubated at 25 °C for 10 min. TCEP was subsequently removed from reduced protein solutions using Amicon Ultra Centrifugal Filters (10 kDa MWCO, Millipore Sigma) in tris-buffered saline (TBS, Sigma Aldrich), and 1 mg/ml antigen was reacted with 5 molar equivalents of pSer-maleimide linkers for 16 h at 4 °C in TBS (pH 7.2–7.4). Free pSer linker was subsequently removed using centrifugal filters in TBS, and pSer-antigen was buffer exchanged to PBS.

pSer_4_-conjugated cytochrome C used for antigenicity profiling of immunogens was prepared as described^16^, using cytochrome C from *Saccharomyces cerevisiae* (Sigma Aldrich). The number of pSer residues conjugated to the antigen was assessed using the Malachite Green Phosphoprotein Phosphate Estimation Assay Kit (Thermo Scientific) against a standard curve of pSer-maleimide linker. The signal from pSer-antigen was compared to the background from an unconjugated antigen control. Fluorescently labeled proteins used in imaging experiments were prepared by reacting 1 mg/ml antigen in 50 mM sodium bicarbonate buffer for 1 h at 25 °C with 6 molar equivalents of AlexaFluor647 NHS ester (Invitrogen) for alum binding studies and whole-mouse imaging. The labeled antigen was purified by centrifugal filtration.

### SMNP adjuvant synthesis

Saponin-MPLA nanoparticles (SMNP) adjuvant was prepared as previously described^23^. Briefly, solutions at 20 mg/ml were prepared of cholesterol, DPPC, and PHAD MPLA (Avanti Polar Lipids) in 20% MEGA-10 detergent (Sigma). Quil-A saponin (InvivoGen) was dissolved in Milli-Q water at a final concentration of 100 mg/ml. These were mixed at a mass ratio of 10:2:1:1 (Quil-A:chol:DPPC:MPLA) and diluted in PBS to a final cholesterol concentration of 1 mg/ml. The solution was equilibrated overnight at 25 °C and then dialyzed against PBS using a 10 kDa MWCO cassette. The adjuvant was then sterile filtered, concentrated using Amicon Ultra Centrifugal Filters (50 kDa MWCO, Millipore Sigma), and purified by FPLC using a Sephacryl S-500 HR size exclusion column (Cytiva Life Sciences). The concentration was determined using a cholesterol quantification assay (Sigma Aldrich).

### Antigen and adjuvant alum binding and release

AlexaFluor647-labeled antigen was loaded onto Alhydrogel (alum, InvivoGen) in TBS at a 1:10 antigen:alum mass ratio, unless otherwise specified, for 30 min on a tube rotator at 25 °C. To assess antigen binding to alum, samples were immediately centrifuged at 10,000 x *g* for 10 min to pellet alum, and the fluorescence of the supernatant was measured against a standard curve of labeled antigen. To assess the release of antigen from alum, mouse serum was added to antigen-alum solutions post-loading to a final mouse serum concentration of 10 vol% and incubated at 37 °C for 24 h unless otherwise specified. Samples were subsequently centrifuged at 10,000 *g* for 10 min to pellet alum, and the fraction of protein bound to alum was measured by fluorescence analysis of the supernatant using a Tecan Infinite M200 Pro plate reader. Experiments investigating SMNP binding and release from alum were performed using Cy7-labeled SMNP, prepared as described incorporating 1,2-distearoyl-*sn*-glycero-3-phosphoethanolamine-*N*-(Cy7) (Avanti Polar Lipids) in place of 10 mol% of the MPLA.

### Antigenicity profiling of immunogens

Antigenicity profiling of antigens was completed by comparing antibody binding curves of pSer-conjugated MD39 on alum against those of unmodified MD39. To capture alum on Nunc Maxisorp ELISA plates (Invitrogen), plates were first coated with pSer_4_-conjugated cytochrome C at 2 μg/ml for 4 h at 25 °C. Alum was then added at 200 μg/ml and captured by pSer_4_-cytochrome C for 16 h at 4 °C. To capture unmodified MD39 with a polyhistidine linker, plates were coated with a rabbit anti-histag antibody (GenScript) at 2 μg/ml for 4 h at 25 °C followed by blocking with 2% BSA in PBS for 16 h at 4 °C. To capture unmodified MD39 expressed with non-polyhistidine linkers, plates were coated with mouse VRC01 at 2 μg/ml for 4 h at 25 °C followed by blocking with 2% BSA in PBS for 16 h at 4 °C. Plates were washed with 0.05% Tween-20 in PBS and incubated with 2 μg/ml protein in 2% BSA in PBS for 2 h at 25 °C. Neutralizing and non-neutralizing antibodies were added at 5 μg/ml with 1:4 serial dilutions for 2 h at 25 °C. Plates were washed, and antibody binding was detected with a goat anti-human HRP conjugated secondary antibody with minimal cross-reactivity (Jackson ImmunoResearch) at 1:5000 dilution in PBS containing 2% BSA and then developed with 3,3′,5,5′-tetramethylbenzidine (ThermoFisher), stopped with 2 N sulfuric acid and immediately read (450 nm with 540 nm reference) on a BioTek Synergy2 plate reader.

### Animals and immunizations

Experiments and handling of mice were conducted under federal, state, and local guidelines under an Institutional Animal Care and Use Committee (IACUC) approved protocol. Female 6–8-week-old BALB/c mice were purchased from the Jackson Laboratory (stock no. 000651). Immunizations were prepared by mixing 10 μg of antigen and 100 μg of alum in 100 μL sterile tris-buffered saline (TBS, Sigma Aldrich) per mouse unless otherwise specified. Antigen was loaded onto alum for 30 min on a tube rotator prior to immunization. When SMNP was added to the immunization, antigen was first loaded onto alum for 30 min on a rotator, after which 5 μg of SMNP, unless otherwise specified, was added into the immunization and incubated with antigen-alum formulations for 30 min prior to immunization. This dose of SMNP corresponds to 5 µg of Quil-A and 0.5 µg MPLA. Mice were immunized subcutaneously at the tail base with 50 μL on each side of the tail base and were subsequently boosted 6 weeks post-prime.

### Antigen-binding and anti-tag ELISAs

Serum was collected from mice retro-orbitally using capillary tubes and stored at −20 °C until analysis. To determine serum IgG titers, Nunc Maxisorp plates (Invitrogen) were coated with a rabbit anti-histag antibody (GenScript) at 2 μg/ml for 4 h at 25 °C in PBS and blocked with 2% BSA in PBS overnight at 4 °C. Plates were washed with 0.05% Tween-20 PBS, and MD39 was added at 2 μg/ml in 2% BSA in PBS for 2 h. Serum dilutions (1:10 dilution followed by 1:50 dilution with 1:4 serial dilutions) were incubated in the plate for 2 h. Plates are washed again, incubated with a goat anti-mouse IgG HRP-conjugated secondary (BioRad) at 1:5000 dilution, and then developed with 3,3′,5,5′-tetramethytlbenzidine (ThermoFisher), stopped with 2 N sulfuric acid, and immediately read (450 nm with 540 nm reference) on a BioTek Synergy2 plate reader. To determine non-trimer base-directed serum IgG titers, Nunc Maxisorp plates (Invitrogen) were coated with a rabbit anti-histag antibody (GenScript) at 2 μg/ml for 4 h at 25 °C in PBS and blocked with 2% BSA in PBS overnight at 4 °C, and then incubated for 30 min with base-binding 12 N antibody. Serum dilutions (1:10 dilution followed by 1:50 dilution with 1:4 serial dilutions) were then added to the plate for 2 h and subsequently followed the protocol for anti-MD39 serum IgG titers. For studies comparing proteins with varying linkers, Nunc Maxisorp plates (Invitrogen) were coated with human VRC01 at 2 μg/ml for 4 h at 25 °C in PBS and blocked with 2% BSA in PBS overnight at 4 °C. Plates were washed with 0.05% Tween-20 PBS, and MD39 with a linker orthogonal to the immunization constructs was added at 2 μg/ml in 2% BSA in PBS for 2 h, and subsequently follow the protocol for anti-MD39 serum IgG titers. To determine anti-PEG serum responses, Nunc Maxisorp plates (Invitrogen) were coated with streptavidin (Invitrogen) at 1 μg/mL for 4 h at 25 °C in PBS and blocked with 2% BSA in PBS overnight at 4 °C. Plates were washed with 5% (w/v) n-Dodecyl-beta-Maltoside (ThermoFisher) in PBS, and biotin-PEG-OH (Creative PEGWorks) was added at 1 μg/ml in 2% BSA in PBS for 2 h. To determine anti-tag serum responses, Nunc Maxisorp plates (Invitrogen) were coated with streptavidin (Invitrogen) at 1 μg/mL for 4 h at 25 °C in PBS and blocked with 2% BSA in PBS overnight at 4 °C. Plates were washed with 0.05% Tween-20 PBS, and biotinylated tags (biotin-HHHHHH, biotin-KKK, and biotin-GGSGGGS; Genscript) were added at 1 μg/ml in 2% BSA in PBS for 2 h. Serum dilutions (1:50 dilution followed by 1:4 serial dilutions) were incubated in the plate for 2 h. Plates are washed again, incubated with a goat anti-mouse IgG or IgM HRP-conjugated secondary (BioRad) at 1:5000 dilution, and then developed with 3,3′,5,5′-tetramethytlbenzidine (ThermoFisher), stopped with 2 N sulfuric acid, and immediately read (450 nm with 540 nm reference) on a BioTek Synergy2 plate reader.

### GC responses

The inguinal lymph nodes were collected from immunized mice 14 days post-immunization unless otherwise specified. For GC analysis, cells were stained for viability (ThermoFisher Live/Dead Fixable Aqua) and against CD3e (BV711, BioLegend, 145-2C11 clone), B220 (PE-Cy7, BioLegend RA3-6B2 clone), CD38 (FITC, BioLegend 90 clone), and GL7 (PerCP-Cy5.5, BioLegend GL7 clone), with antigen-specific staining completed using biotinylated MD39 conjugated to streptavidin-BV421 (BioLegend) and streptavidin-PE (BioLegend). Samples were analyzed by flow cytometry on a BD Celesta and analyzed on FlowJo.

### Whole-mouse imaging of vaccination drainage

Mice were immunized subcutaneously at the tail base with fluorescently labeled antigens. Immunizations were prepared as described, using fluorescently labeled components as indicated. For studies including fluorescently labeled components, immunizations were prepared by loading antigen onto alum in sterile tris-buffered saline (TBS, Sigma Aldrich) for 30 min on a tube rotator prior to adding SMNP if indicated and incubating for 30 min on a tube rotator. Imaging was completed using a PerkinElmer Xenogen Spectrum in vivo imaging system, and the fluorescent signal at the injection site was quantified using LivingImage software. The radiant efficiency was tracked longitudinally to monitor drainage from the injection site.

### Statistical analysis

All data were plotted, and all statistical analyses were performed using GraphPad Prism 9 software (La Jolla, CA). All graphs display mean values, and the error bars represent the standard deviation unless otherwise specified. No samples or animals were excluded from the analyses. Statistical comparison was performed using a Mann–Whitney *U* test for single timepoint/factor data with two groups or a one-way ANOVA followed by Tukey’s or Dunnett’s multiple comparison tests for single timepoint/factor data with greater than two groups. A two-way ANOVA followed by Tukey’s or Sidak’s multiple comparison tests was used for multi-timepoint longitudinal data. Statistical analysis of antibody titer was completed using log-transformed data. Data were considered statistically significant if the *p*-value was less than 0.05.

### Reporting summary

Further information on research design is available in the Nature Research Reporting Summary linked to this article.

### Supplementary information

Supplementary Materials

REPORTING SUMMARY

## Data Availability

All data generated in this study are available from the corresponding author upon reasonable request.
