# Supplementary material for: Optimization of an alum-anchored clinical HIV vaccine candidate
Source: NPJ Vaccines. 2023 Aug 12;8:117. doi: 10.1038/s41541-023-00711-0 (PMC10423202; doi:10.1038/s41541-023-00711-0)
Supplement: Supplementary file 1 — Supplementary Materials [file 41541_2023_711_MOESM1_ESM.pdf]

SUPPLEMENTARY MATERIALS

Optimization of an alum-anchored clinical HIV vaccine candidate

Kristen A. Rodrigues[1,2,3,4], Christopher A. Cottrell[4,5], Jon M. Steichen[4,6,7], Bettina Groschel[4,6,7], Wuhbet Abraham[1,3], Heikyung Suh[1,3], Yash Agarwal[1,8], Kaiyuan Ni[1,3,4], Jason Y. H. Chang[1,3], Parisa Yousefpour[1], Mariane B. Melo[1,3,4], William R. Schief[3,4,6,7]*, Darrell J. Irvine[1,3,4,8,9,10,11]*

Affiliations

[1]Koch Institute for Integrative Cancer Research, Massachusetts Institute of Technology, Cambridge, MA 02139 USA.

[2]Harvard-MIT Health Sciences and Technology Program, Institute for Medical Engineering and Science, Massachusetts Institute of Technology, Cambridge, MA 02139 USA.

[3]Ragon Institute of Massachusetts General Hospital, Massachusetts Institute of Technology and Harvard University, Cambridge, MA 02139 USA.

[4]Consortium for HIV/AIDS Vaccine Development, The Scripps Research Institute, La Jolla, CA 92037 USA.

[5]Department of Integrative Structural and Computational Biology, The Scripps Research Institute, La Jolla, CA 92037 USA.

[6]Department of Immunology and Microbiology, The Scripps Research Institute, La Jolla, CA 92037 USA.

[7]IAVI Neutralizing Antibody Center, The Scripps Research Institute, La Jolla, CA 92037 USA.

[8]Department of Biological Engineering, Massachusetts Institute of Technology, Cambridge, MA 02139 USA.

[9]Department of Chemical Engineering, Massachusetts Institute of Technology, Cambridge, MA 02139 USA.

[10]Department of Materials Science and Engineering, Massachusetts Institute of Technology, Cambridge, MA 02139 USA.

[11]Howard Hughes Medical Institute, Chevy Chase, MD 20815 USA.

*Correspondence: William R. Schief (schief@scripps.edu), Darrell J. Irvine (djirvine@mit.edu)

TABLE

Supplementary Table 1: Sequence of immunogen, with C-terminal linker sequence shown red and free cysteine shown in blue.

**MD39_his with C-terminal cysteine**
ETGAENLWVTVYYGVPVWKDAETTLFCASDAKAYETEKHNVWATHACVPTDPNPQEIHLENVTEEF<br>NMWKNNMVEQMHEDIISLWDQSLKPCVKLTPLCVTLQCTNVTNNITDDMRGELKNCSFNMTTELRD<br>KKQKVYSLFYRLDVVQINENQGNRSNNSNKEYRLINCNTSAITQACPKVSFEPIPIHYCAPAGFAILKC<br>KDKKFNGTGPCPSVSTVQCTHGIKPVVSTQLLLNGSLAEEEVIIRSENITNNAKNILVQLNTPVQINCT<br>RPNNNTVKSIRIGPGQAFYYTGDIIGDIRQAHCNVSKATWNETLGKVVKQLRKHFGNNTIIRFAQSSG<br>GDLEVTTHSFNCGGEFFYCNTSGLFNSTWISNTSVQGSNSTGSNDSITLPCRIKQIINMWQRIGQAM<br>YAPPIQGVIRCVSNITGLILTRDGGSTNSTTETFRPGGGDMRDNWRSELYKYKVVKIEPLGVAPTRCK<br>RRVVGRRRRRRAVGIGAVSLGFLGAAGSTMGAASMTLTVQARNLLSGIVQQQSNLLRAPEPQQHLL<br>KDTHWGIKQLQARVLAVEHYLRDQQLLGIWGCSGKLICCTNVPWNSSWSNRNLSEIWDNMTWLQW<br>DKEISNYTQIIYGLLEESQNQQEKNEQDLLALDGTKHHHHHHC

FIGURES

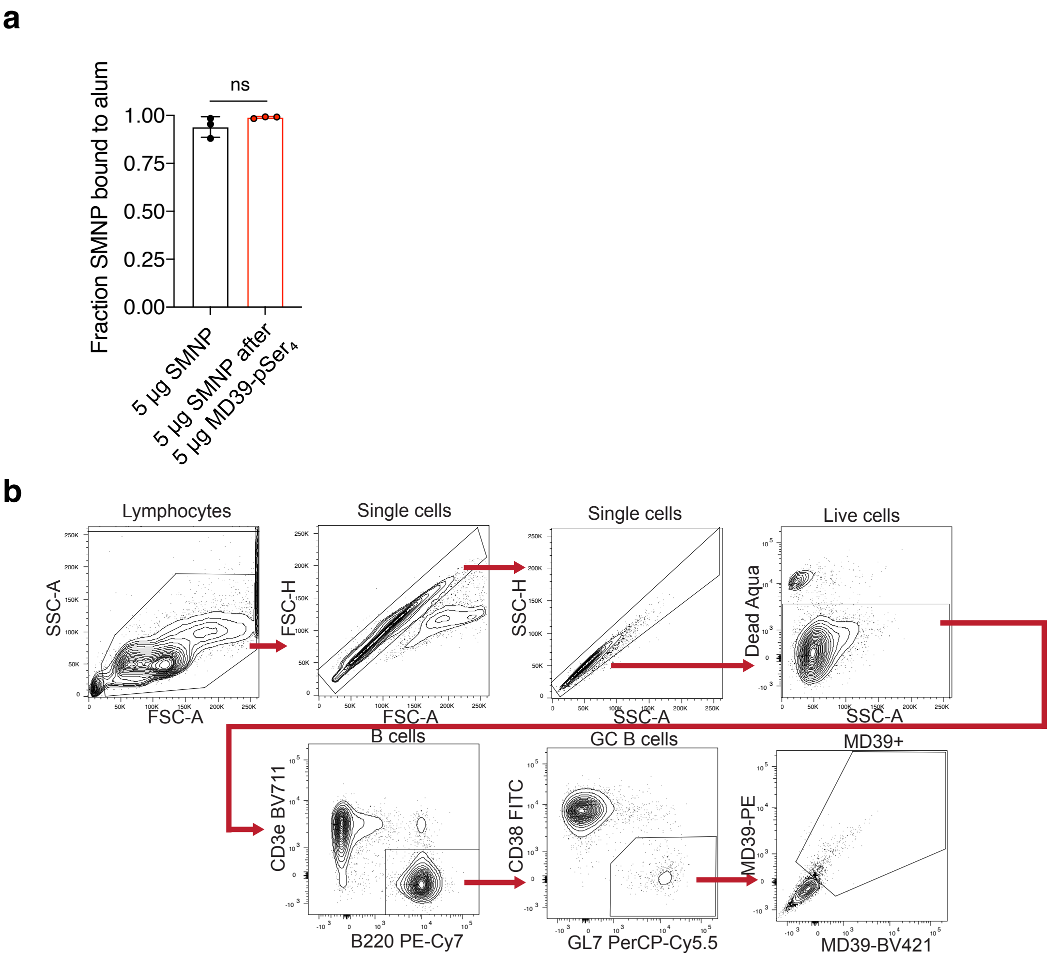

Supplementary Figure 1. SMNP exhibits strong binding to alum. a Saponin/phospholipid nanoparticle adjuvant (SMNP) was mixed with alum with or without MD39-pSer$_4$, and the fraction of SMNP bound to alum was assessed after incubation for 24 hours in 10% mouse serum at 37°C. Values plotted are means ± standard deviation. Statistical significance was determined by Mann-Whitney U test. ns p>0.05. **b** Representative flow cytometry gating approach for MD39-specific germinal center (GC) B cells.

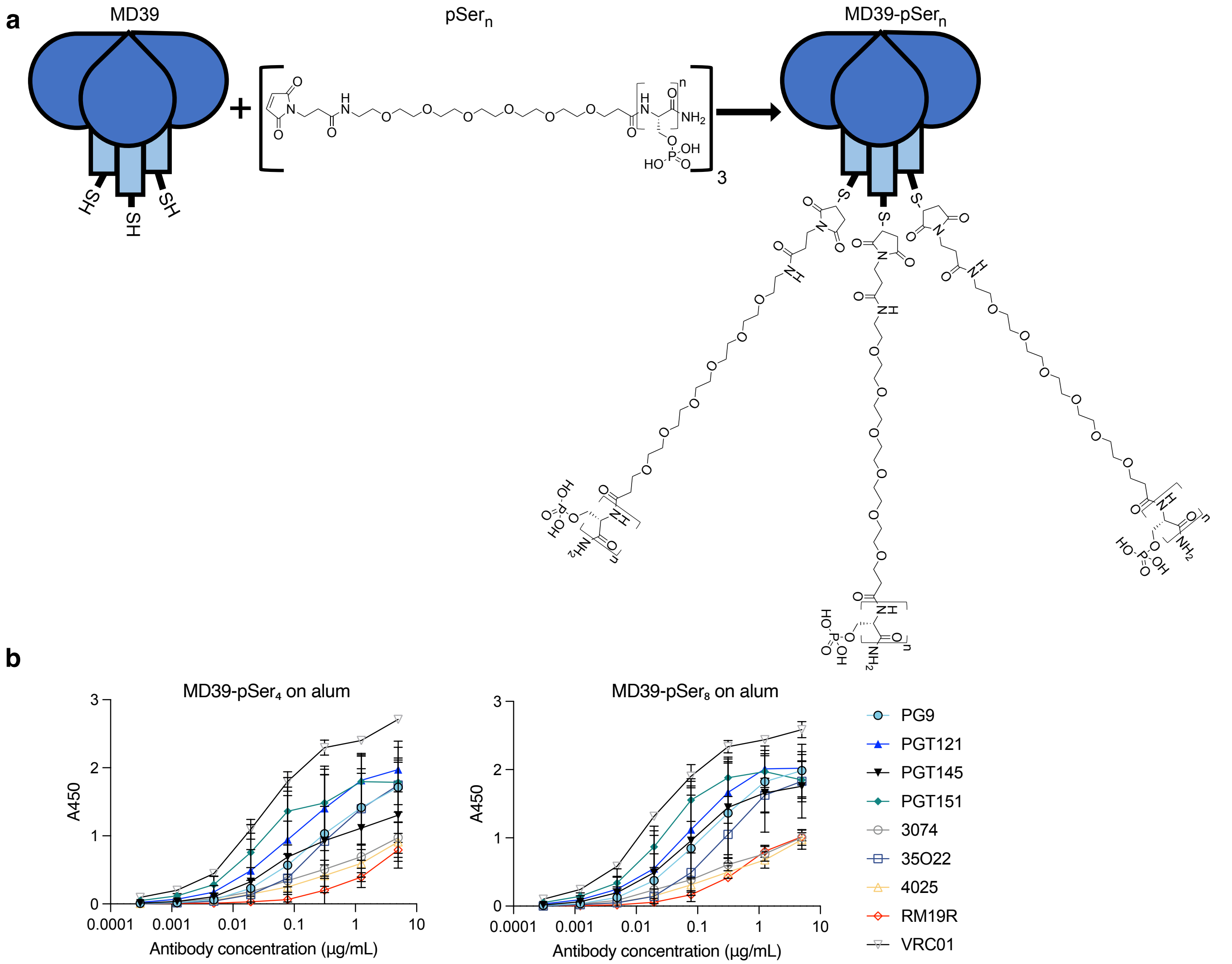

Supplementary Figure 2. Phosphoserine-conjugated MD39 retains the physical properties of MD39. a Chemical structure of phosphoserine (pSer) peptide containing a maleimide group linked to a 6-unit poly(ethylene glycol) spacer followed by repeat pSer residues. Thiol-maleimide coupling mediates pSer-conjugation of MD39 trimers. **b** Antigenicity profiling of MD39-pSer$_4$ and MD39-pSer$_8$ captured on alum. Shown are binding profiles of antibodies (*n*=3 replicates). Values plotted are means ± standard deviation.

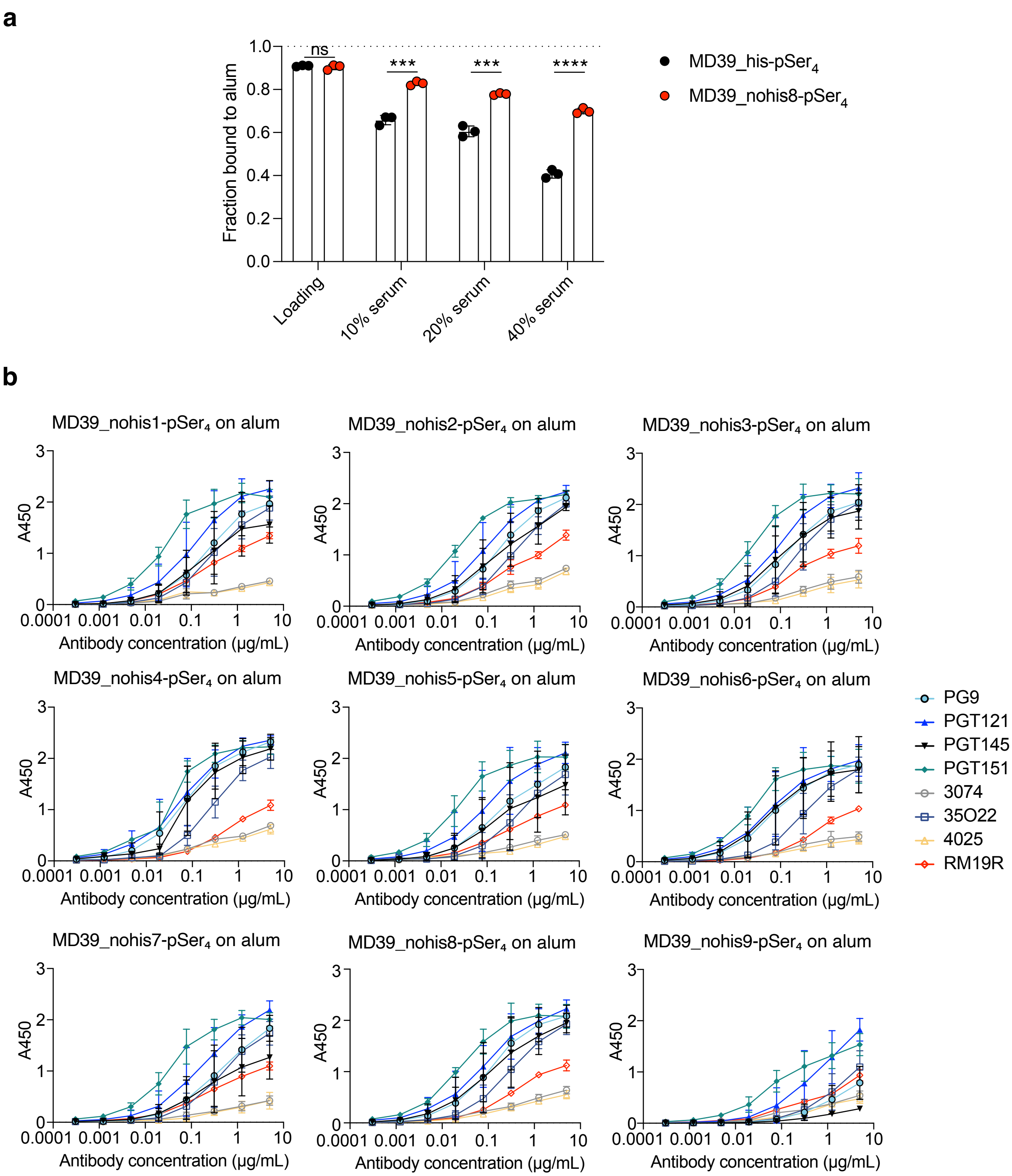

Supplementary Figure 3. Phosphoserine-conjugated MD39 constructs with alternate linkers exhibit strong alum binding and retain the antigenicity profile of MD39. a pSer-conjugated MD39 constructs were mixed with alum, and the fraction of protein bound to alum was assessed before ("Loading") and after incubation for 24 hours in varying percentages of mouse serum at 37°C. Values plotted are means ± standard deviation. Statistical significance was determined by unpaired Student's t-test. **b** Antigenicity profiling of MD39-pSer$_4$ captured on alum (*n*=3 replicates). Values plotted are means ± standard deviation. ns p>0.05, **** p<0.0001.

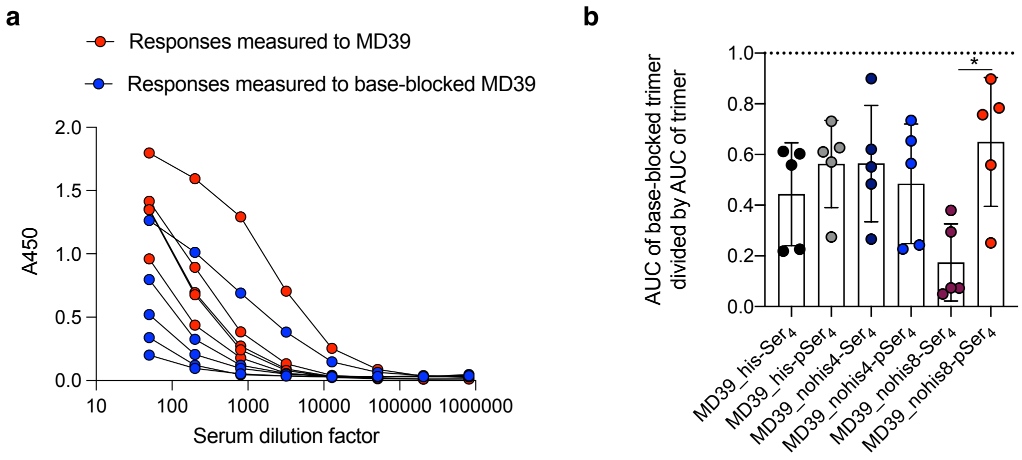

Supplementary Figure 4. pSer-conjugated MD39 constructs with alternate linkers elicit strong humoral immune responses against vaccine-relevant epitopes. BALB/c mice (*n*=5 animals/group) were immunized with 10 µg Ser_4- or $pSer_4$- conjugated MD39 constructs and 100 µg alum and boosted with 5 µg Ser_4- or $pSer_4$- conjugated MD39 constructs and 50 µg alum with 5 µg SMNP at 6 weeks, and serum IgG responses were assessed by ELISA at day 56. **a** Shown are absorbance curves for MD39_his-Ser_4 immunized mice measured against MD39 and MD39 base-blocked with 12N antibody. **b** Plotted are the fraction of integrated absorbance curves retained in ELISAs completed in the presence of base-blocking 12N antibody. Dashed line indicates value for which there is no MD39 base-directed response. Statistical significance was determined by one-way ANOVA followed by Tukey's multiple comparison test. Values plotted are means ± standard deviation. ns $p>0.05$, * $p<0.05$.

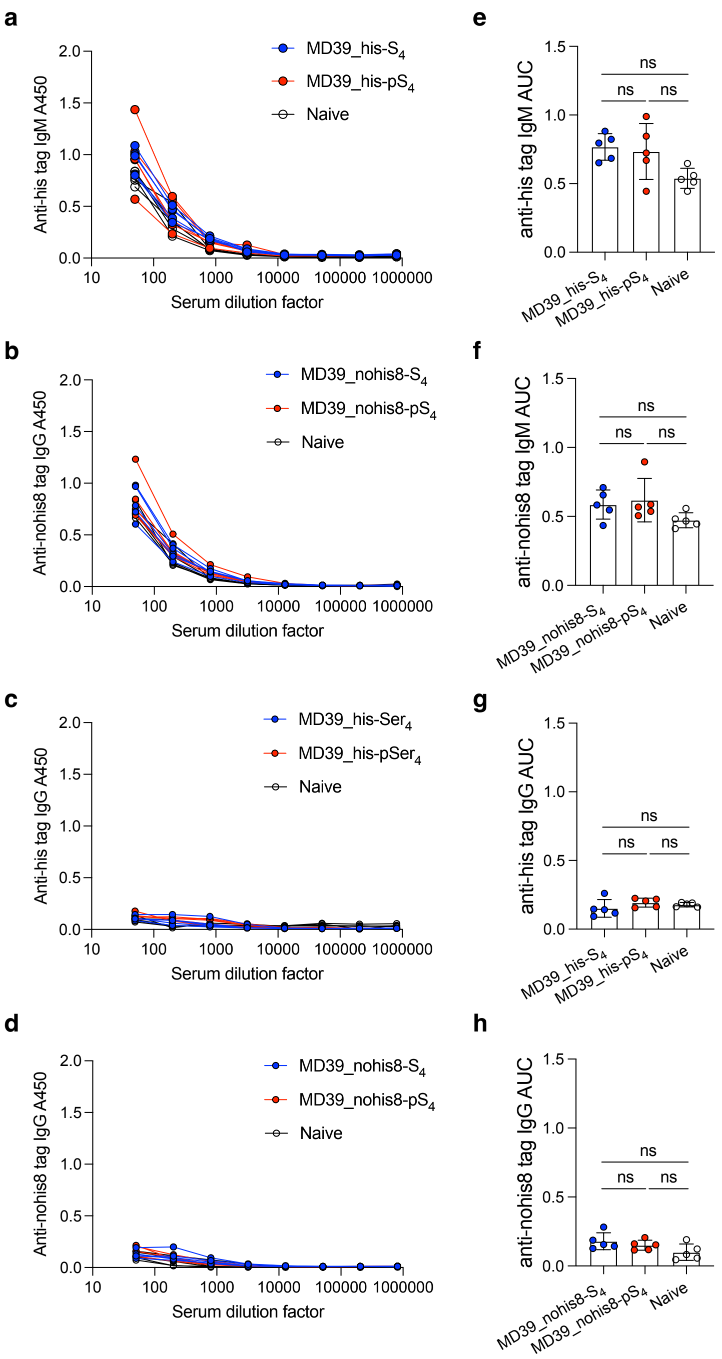

Supplementary Figure 5. MD39 constructs with alternate linkers elicit no detectable serum IgM or IgG responses above naïve baseline. BALB/c mice (*n*=5 animals/group) were immunized with 10 μg Ser_4- or $pSer_4$- conjugated MD39 constructs and 100 μg alum and boosted with 5 μg Ser_4- or $pSer_4$-conjugated MD39 constructs and 50 μg alum with 5 μg SMNP at 6 weeks, and serum IgG responses were assessed at day 56 by ELISA against biotinylated HHHHHH (his) or KKK (nohis8) peptides captured on plates coated with streptavidin. **a** Anti-his tag IgM, **b** anti-nohis8 tag IgM, **c** anti-his tag IgG, and **d** anti-nohis8 tag IgG. Shown are individual mouse ELISA curves, which are calculated as area under the curve (AUC) in **e-h**. Values plotted are means ± standard deviation. Statistical significance was determined by one-way ANOVA followed by Sidak's multiple comparisons test. ns p>0.05, ** p<0.01.

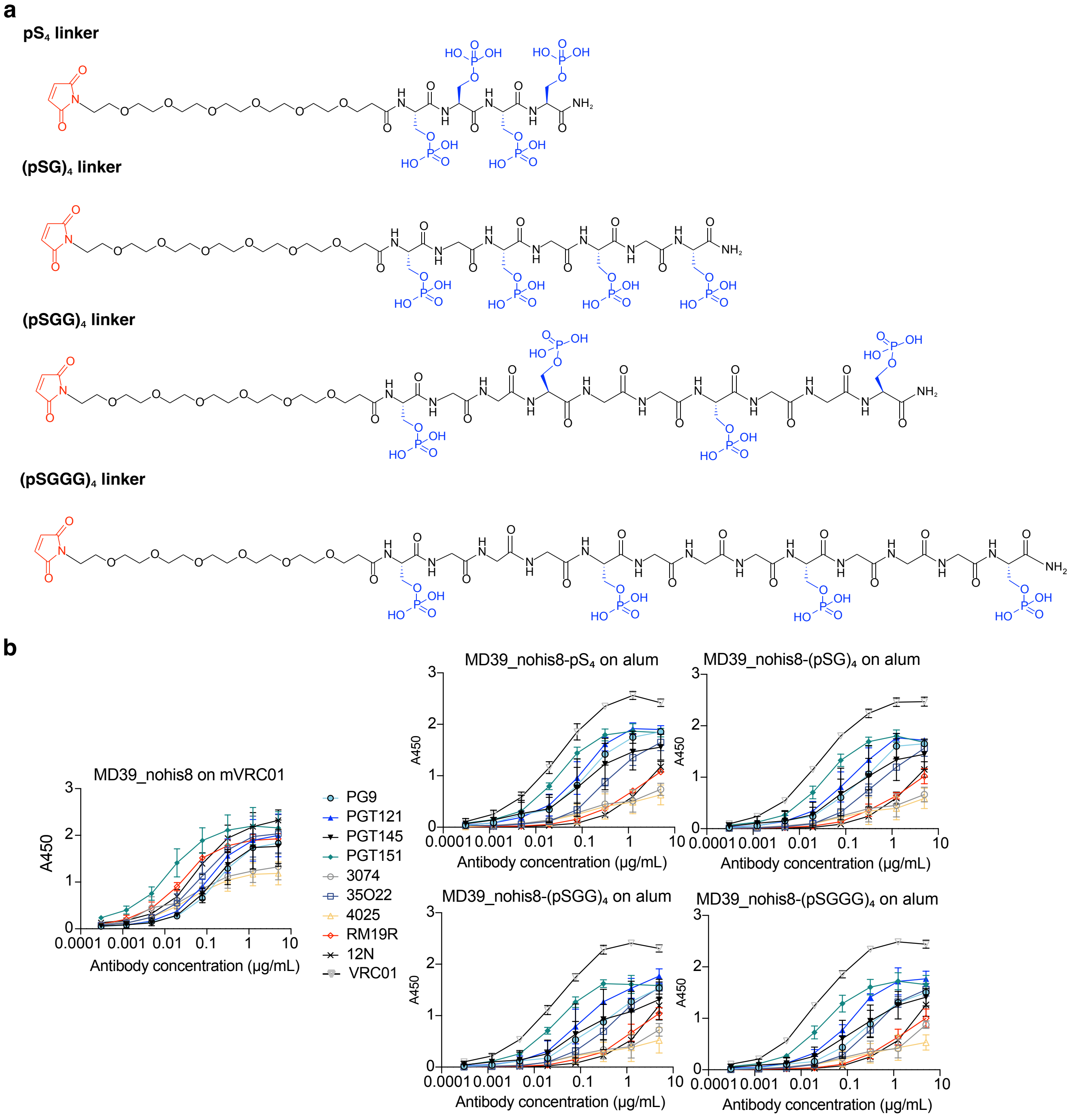

Supplementary Figure 6. Glycine spacers between pSer residues do not significantly alter the antigenicity profile of MD39-pSer₄. a Chemical structures of pSer linkers containing 0-3 glycine spacers (pS_4, $(pSG)_4$, $(pSGG)_4$, and $(pSGG)_4$, respectively). **b** Antigenicity profiling of unmodified or pSer-conjugated MD39_nohis8 (n=3 replicates). Values plotted are means ± standard deviation.

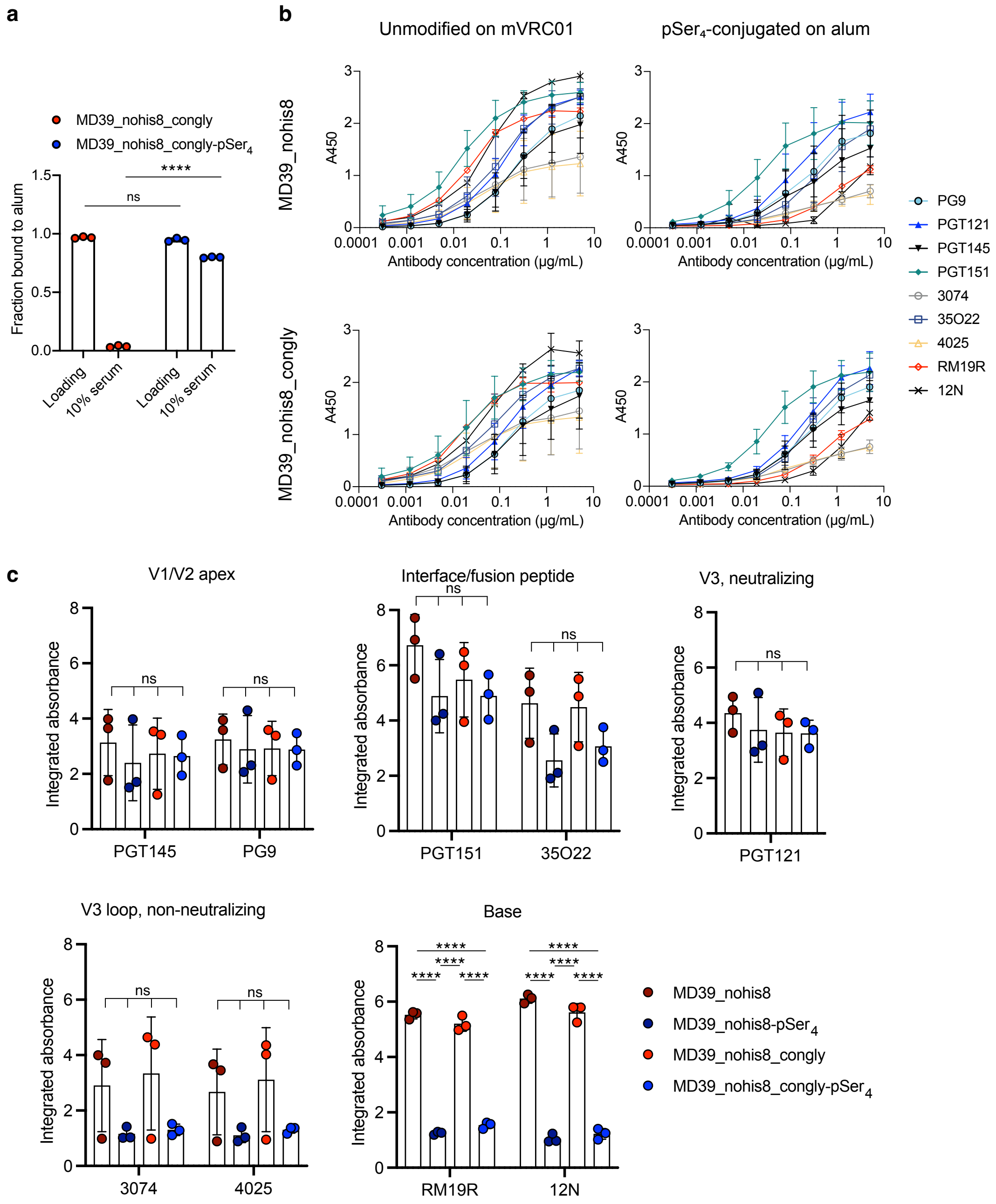

Supplementary Figure 7. Glycan hole filled MD39 exhibits strong alum binding and retains antigenicity profile when conjugated to pSer$_4$. a pSer-conjugated or unmodified MD39 constructs were mixed with alum, and the fraction of protein bound to alum was assessed before (“Loading”) and after incubation for 24 hours in 10% mouse serum at 37°C. Values plotted are means ± standard deviation. Statistical significance was determined by one-way ANOVA followed by Tukey’s multiple comparison test. **b** Antigenicity profiling of MD39-pSer$_4$ with and without a filled glycan hole (congly) on alum compared to unmodified MD39 captured by mVRC01. Values plotted are means ± standard deviation. **c** Antigenicity profiling of unmodified or pSer-conjugated MD39 with and without a filled glycan hole (*n*=3 replicates). Shown are the area under individual binding curves. Statistical significance was determined by one-way ANOVA followed by Tukey’s multiple comparison test. ns $p>0.05$, * $p<0.05$, **** $p<0.0001$.

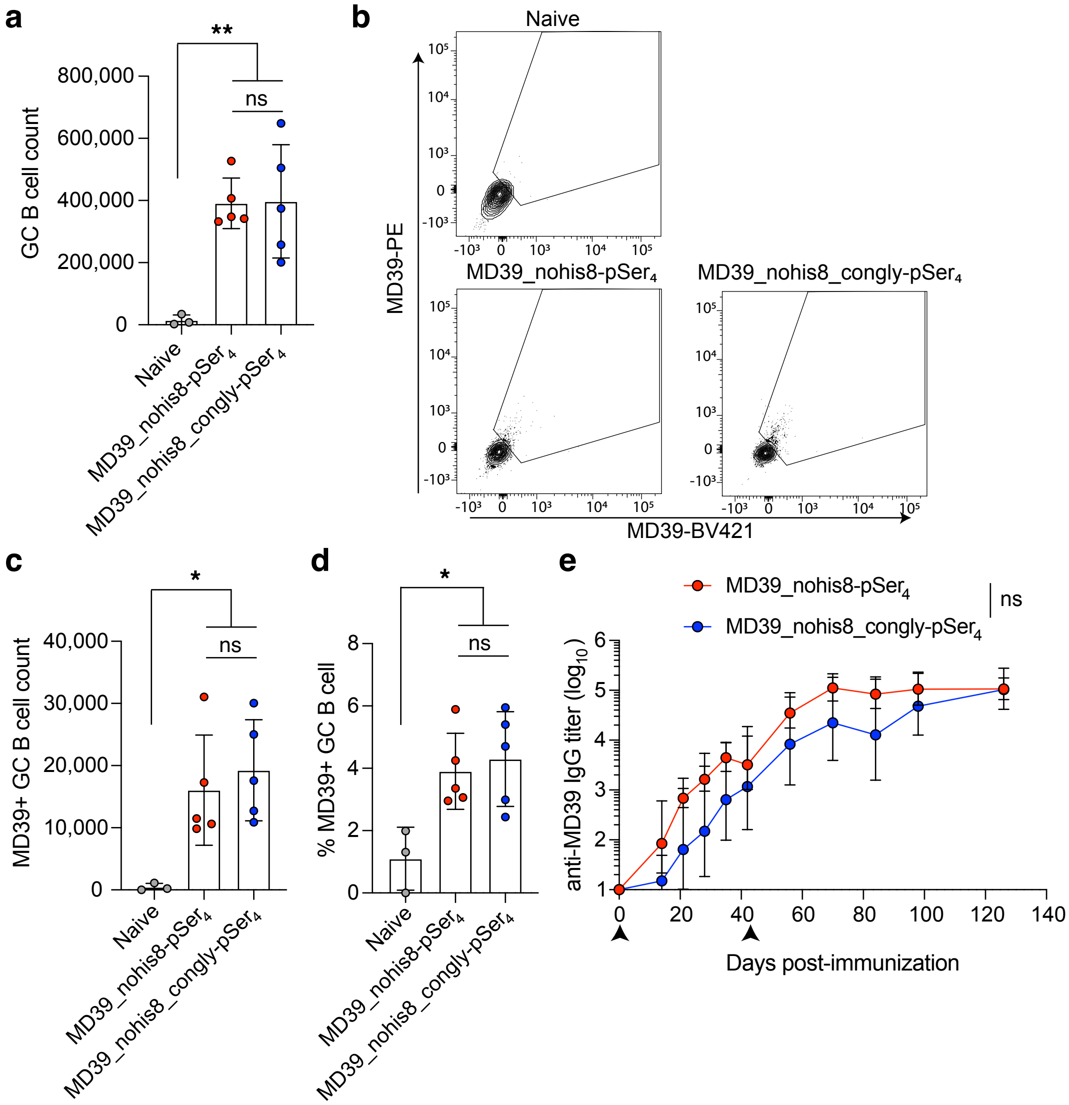

Supplementary Figure 8. MD39-pSer construct with a filled glycan hole (congly) elicits strong humoral immune responses. a BALB/c mice were immunized with 5 µg pSer$_4$-conjugated MD39 constructs and 50 µg alum with 5 µg SMNP, and germinal center (GC) B cell responses in draining inguinal lymph nodes were analyzed by flow cytometry 14 days post-immunization. Values plotted are means ± standard deviation. Statistical significance was determined by one-way ANOVA followed by Tukey's multiple comparison test. **b** Shown are representative flow cytometry gating plots of MD39-specific GC B cell analysis, plotted by MD39-specific GC B cell count in **c** and the percentage of GC B cells that are MD39-specific in **d**. Values plotted are means ± standard deviation. Statistical significance was determined by one-way ANOVA followed by Tukey's multiple comparison test. **e** Mice (n=5 animals/group) were immunized with 10 µg pSer$_4$-conjugated MD39 constructs and 100 µg alum and boosted with 5 µg Ser$_4$- or pSer$_4$- conjugated MD39 constructs and 50 µg alum with 5 µg SMNP at 6 weeks, and serum IgG responses were assessed longitudinally by ELISA. Arrows indicate immunizations. Values plotted are geometric means ± geometric standard deviation. Statistical significance was determined by two-way ANOVA followed by Sidak's multiple comparison test. ns $p>0.05$, * $p<0.05$, ** $p<0.01$, *** $p<0.001$, **** $p<0.0001$.

a

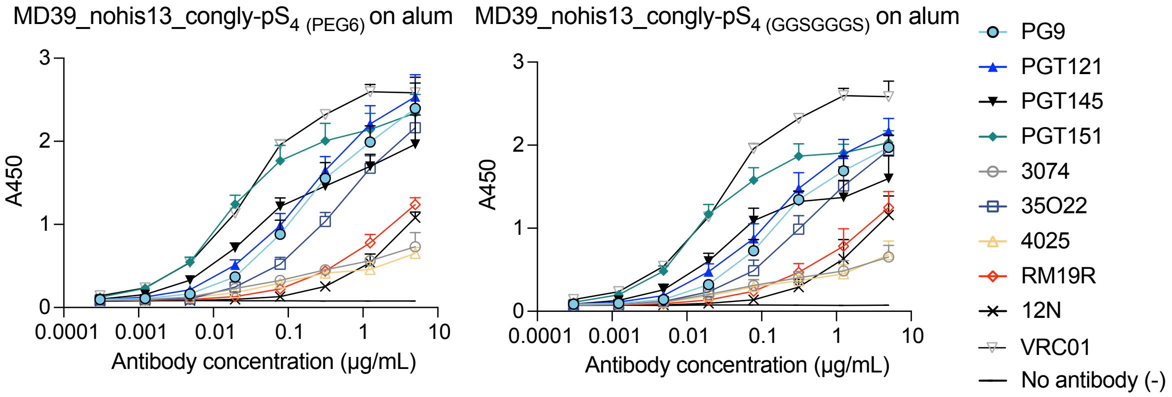

Supplementary Figure 9. Replacing the 6-unit PEG spacer with a flexible glycine/serine spacer does not significantly alter the antigenicity profile of MD39-pSer. a Antigenicity profiling of MD39-pSer$_4$ with either PEG$_6$ or GGSGGGS spacer (n=3 replicates). Values plotted are means ± standard deviation.

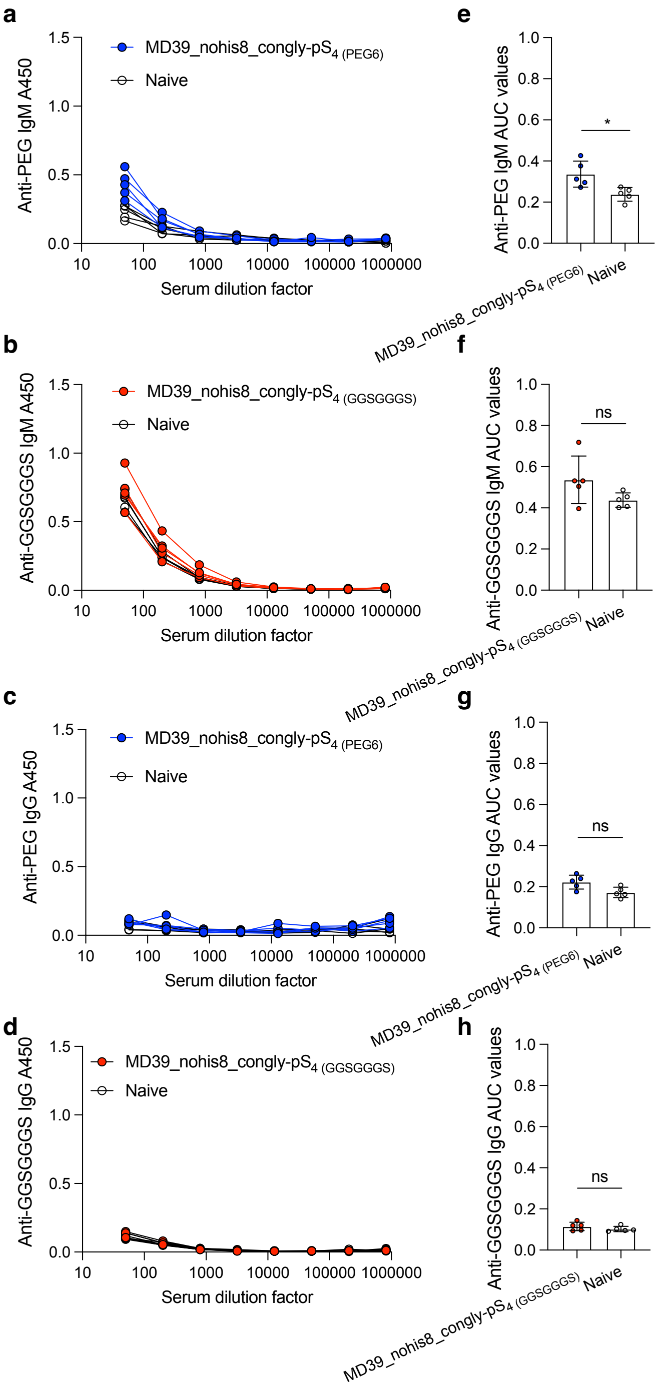

Supplementary Figure 10. MD39 constructs conjugated to pSer linkers elicit weak anti-PEG serum IgM responses. BALB/c mice (*n*=5 animals/group) were immunized with 10 µg Ser_4- or $pSer_4$-conjugated MD39 constructs and 100 µg alum and boosted with 5 µg Ser_4- or $pSer_4$- conjugated MD39 constructs and 50 µg alum with 5 µg SMNP at 6 weeks, and serum IgG responses were assessed at day 56 by ELISA against biotinylated PEG_6 or GGSGGGS peptides captured on plates coated with streptavidin. **a** Anti-PEG IgM, **b** anti-GGSGGGS IgM, **c** anti-PEG IgG, and **d** anti-GGSGGGS IgG. Shown are individual mouse ELISA curves, which are calculated as area under the curve (AUC) in **e-h**. Values plotted are means ± standard deviation. Statistical significance was determined by unpaired Student's t-test. ns $p>0.05$, * $p<0.05$.

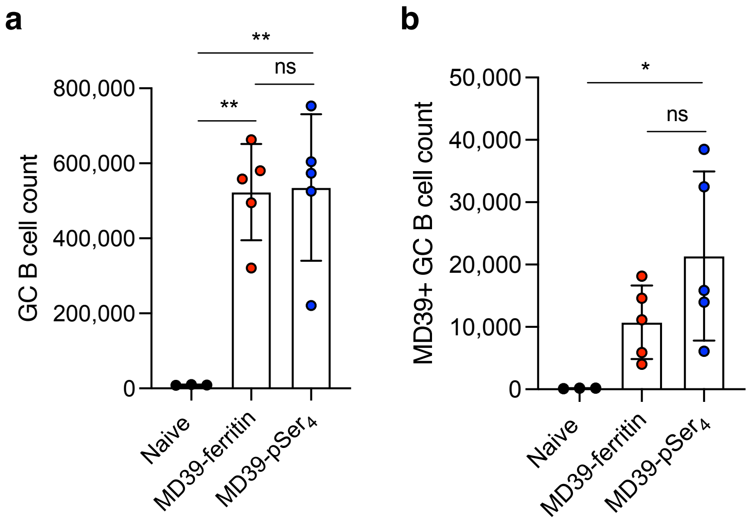

Supplementary Figure 11. Nanoparticle formulated MD39-ferritin and MD39-pSer elicit comparable humoral immune responses. a BALB/c mice (*n*=5 animals/group) were immunized with 5 µg equivalent of MD39 and 50 µg alum combined with 5 µg SMNP, and germinal center (GC) B cell responses in draining inguinal lymph nodes were analyzed by flow cytometry 14 days post-immunization. **b** MD39-specific GC B cell counts. Values plotted are means ± standard deviation. Statistical significance was determined by one-way ANOVA followed by Tukey's multiple comparison test. ns p>0.05, * p<0.05, ** p<0.01.
