# Supplementary material for: Optimization of an alum-anchored clinical HIV vaccine candidate
Source: NPJ Vaccines. 2023 Aug 12;8:117. doi: 10.1038/s41541-023-00711-0 (PMC10423202; doi:10.1038/s41541-023-00711-0)
Supplement: Supplementary file 2 — REPORTING SUMMARY [file 41541_2023_711_MOESM2_ESM.pdf]

nature portfolio

Corresponding author(s): William R. Schief; Darrell J. Irvine

Last updated by author(s): Jun 22, 2023

Reporting Summary

Nature Portfolio wishes to improve the reproducibility of the work that we publish. This form provides structure for consistency and transparency in reporting. For further information on Nature Portfolio policies, see our Editorial Policies and the Editorial Policy Checklist.

Statistics

For all statistical analyses, confirm that the following items are present in the figure legend, table legend, main text, or Methods section.

n/a	Confirmed	
☐	☒	The exact sample size (*n*) for each experimental group/condition, given as a discrete number and unit of measurement
☐	☒	A statement on whether measurements were taken from distinct samples or whether the same sample was measured repeatedly
☐	☒	The statistical test(s) used AND whether they are one- or two-sided<br>*Only common tests should be described solely by name; describe more complex techniques in the Methods section.*
☒	☐	A description of all covariates tested
☐	☒	A description of any assumptions or corrections, such as tests of normality and adjustment for multiple comparisons
☐	☒	A full description of the statistical parameters including central tendency (e.g. means) or other basic estimates (e.g. regression coefficient) AND variation (e.g. standard deviation) or associated estimates of uncertainty (e.g. confidence intervals)
☐	☒	For null hypothesis testing, the test statistic (e.g. *F*, *t*, *r*) with confidence intervals, effect sizes, degrees of freedom and *P* value noted<br>*Give P values as exact values whenever suitable.*
☒	☐	For Bayesian analysis, information on the choice of priors and Markov chain Monte Carlo settings
☒	☐	For hierarchical and complex designs, identification of the appropriate level for tests and full reporting of outcomes
☒	☐	Estimates of effect sizes (e.g. Cohen's *d*, Pearson's *r*), indicating how they were calculated

Our web collection on statistics for biologists contains articles on many of the points above.

Software and code

Policy information about availability of computer code

Data collection	Flow cytometry data were obtained using BD FACSDiva software and analyzed on FlowJo. ELISAs and fluorescence assays were measured using a BioTek Synergy2 plate reader or Tecan Infinite M200 Pro plate reader. In vivo IVIS imaging was completed using a PerkinElmer Xenogen Spectrum in vivo imaging system, and the fluorescent signal at the injection site was quantified using LivingImage software. All data were plotted and all statistical analyses were performed using GraphPad Prism 9 software.
Data analysis	Flow cytometry data were analyzed in FlowJo 10.7.1. All data were plotted and all statistical analyses were performed using GraphPad Prism 9.3.1. Microsoft Office (version 16.69) and Adobe Illustrator (version 25.2) were used to draft the manuscript. IVIS data were analyzed using LivingImage version 4.5.

For manuscripts utilizing custom algorithms or software that are central to the research but not yet described in published literature, software must be made available to editors and reviewers. We strongly encourage code deposition in a community repository (e.g. GitHub). See the Nature Portfolio guidelines for submitting code & software for further information.

Data

Policy information about availability of data

All manuscripts must include a data availability statement. This statement should provide the following information, where applicable:

- Accession codes, unique identifiers, or web links for publicly available datasets
- A description of any restrictions on data availability
- For clinical datasets or third party data, please ensure that the statement adheres to our policy

All data needed to evaluate the conclusions in the paper are present in the paper and/or the Supplementary Materials. All data generated in this study are available from the corresponding author upon reasonable request.

Research involving human participants, their data, or biological material

Policy information about studies with human participants or human data. See also policy information about sex, gender (identity/presentation), and sexual orientation and race, ethnicity and racism.

Reporting on sex and gender	No human participants, their data, or their biological material was used in these studies.
Reporting on race, ethnicity, or other socially relevant groupings	No human participants, their data, or their biological material was used in these studies.
Population characteristics	No human participants, their data, or their biological material was used in these studies.
Recruitment	No human participants, their data, or their biological material was used in these studies.
Ethics oversight	No human participants, their data, or their biological material was used in these studies.

Note that full information on the approval of the study protocol must also be provided in the manuscript.

Field-specific reporting

Please select the one below that is the best fit for your research. If you are not sure, read the appropriate sections before making your selection.

☒ Life sciences ☐ Behavioural & social sciences ☐ Ecological, evolutionary & environmental sciences

For a reference copy of the document with all sections, see nature.com/documents/nr-reporting-summary-flat.pdf

Life sciences study design

All studies must disclose on these points even when the disclosure is negative.

Sample size	Sample sizes were determined based on prior experiments to obtain statistically significant data.
Data exclusions	No data or mice were excluded.
Replication	All attempts at replication were successful. All in vitro and in vivo studies were completed with the reported number of biological replicates. Antigenicity ELISAs were pooled across two independent experiments. Where n>10 for in vivo mouse studies, the data represent two independent experiments.
Randomization	Mice were randomized into groups.
Blinding	No blinding was used during the studies.

Reporting for specific materials, systems and methods

We require information from authors about some types of materials, experimental systems and methods used in many studies. Here, indicate whether each material, system or method listed is relevant to your study. If you are not sure if a list item applies to your research, read the appropriate section before selecting a response.

Materials & experimental systems

n/a	Involved in the study	
☐	☒	Antibodies
☐	☒	Eukaryotic cell lines
☒	☐	Palaeontology and archaeology
☐	☒	Animals and other organisms
☒	☐	Clinical data
☒	☐	Dual use research of concern
☒	☐	Plants

Methods

n/a	Involved in the study	
☒	☐	ChIP-seq
☐	☒	Flow cytometry
☒	☐	MRI-based neuroimaging

Antibodies

Antibodies used	Flow Cytometry: CD3e (BV711, 145-2C11 clone; BioLegend, 100349), B220 (PE-Cy7, RA3-6B2 clone; BioLegend, catalog no. 103221), CD38 (FITC, BioLegend 90 clone, catalog no. 102705), and GL7 (PerCP-Cy5.5, BioLegend GL7 clone, catalog no. 144609), with antigen specific staining completed using biotinylated MD39 conjugated to streptavidin-BV421 (BioLegend, catalog no. 405226) and streptavidin-PE (BioLegend, catalog no. 405203).<br><br>Antigenicity ELISAs: Antibodies used for antigenicity profiling of immunogens were obtained from the NIH.
Validation	Antibodies were validated by the manufacturer (BioLegend or NIH).

Eukaryotic cell lines

Policy information about cell lines and Sex and Gender in Research

Cell line source(s)	FreeStyle 293-F cells (ThermoFisher)
Authentication	Each cell line was maintained separately and stocked in early passages, to minimize contamination and to preserve cell identity.
Mycoplasma contamination	Cell lines were confirmed to be absent of mycoplasma contamination by PCR.
Commonly misidentified lines (See ICLAC register)	No commonly misidentified cell lines were used in these studies.

Animals and other research organisms

Policy information about studies involving animals; ARRIVE guidelines recommended for reporting animal research, and Sex and Gender in Research

Laboratory animals	Female BALB/c (JAX, 000651) mice at 6-10 weeks age (~20 g) were purchased and maintained in the animal facility at Massachusetts Institute of Technology.
Wild animals	No wild animals were used in these studies.
Reporting on sex	Female BALB/c (JAX, 000651) mice were used in these studies.
Field-collected samples	No field-collected samples were used in these studies.
Ethics oversight	All animal studies and procedures were carried out following federal, state and local guidelines under an animal protocol approved by the institutional animal care and use committee at Massachusetts Institute of Technology.

Note that full information on the approval of the study protocol must also be provided in the manuscript.

Flow Cytometry

Plots

Confirm that:

☒ The axis labels state the marker and fluorochrome used (e.g. CD4-FITC).

☒ The axis scales are clearly visible. Include numbers along axes only for bottom left plot of group (a 'group' is an analysis of identical markers).

☒ All plots are contour plots with outliers or pseudocolor plots.

☒ A numerical value for number of cells or percentage (with statistics) is provided.

Methodology

Sample preparation	The inguinal lymph nodes were collected from immunized mice 14 days after immunization unless otherwise specified. Draining lymph nodes were mechanically processed into single cell suspensions and filtered using a 70 um filter. For germinal center analysis, cells were stained for viability (Thermo Fisher Scientific Live/ Dead Fixable Aqua, catalog no. L34957) and subsequently against CD3e (BV711, 145-2C11 clone; BioLegend, 100349), B220 (PE-Cy7, RA3-6B2 clone; BioLegend, catalog no. 103221), CD38 (FITC, 90 clone; Bio- Legend, catalog no. 102705), and GL7 (PerCP-Cy5.5, GL7 clone; BioLegend, catalog no. 144609), with antigen-specific staining completed using biotinylated MD39 conjugated to streptavidin-BV421 (BioLegend, catalog no. 405226) and streptavidin-PE (phycoerythrin) (BioLegend, catalog no. 405203).
Instrument	Data were collected on a BD Celesta flow cytometer.
Software	Data were collected using BD FACSDiva (BD Biosciences) and analyzed in FlowJo 10.7.1. All data were plotted and all statistical analyses were performed using GraphPad Prism 9.3.1.
Cell population abundance	No cell sorting was used in these studies.
Gating strategy	Gating for MD39-specific germinal center B cells is presented in Supplementary Fig. 1b.

☒ Tick this box to confirm that a figure exemplifying the gating strategy is provided in the Supplementary Information.
